# Proteomic mapping of differentially vulnerable pre-synaptic populations identifies regulators of neuronal stability *in vivo*

**DOI:** 10.1038/s41598-017-12603-0

**Published:** 2017-09-29

**Authors:** Maica Llavero Hurtado, Heidi R. Fuller, Andrew M. S. Wong, Samantha L. Eaton, Thomas H. Gillingwater, Giuseppa Pennetta, Jonathan D. Cooper, Thomas M. Wishart

**Affiliations:** 1 Division of Neurobiology, The Roslin Institute and Royal (Dick) School of Veterinary Studies, University of Edinburgh, Easter Bush, Midlothian, EH25 9RG UK; 2 Institute for Science and Technology in Medicine, Keele University, Staffordshire, Keele, ST5 5BG UK; 3 Department of Basic and Clinical Neuroscience, Maurice Wohl Clinical Neuroscience Institute, Institute of Psychiatry, Psychology & Neuroscience, King’s College London, London, SE5 9RX UK; 4 Centre for Integrative Physiology, University of Edinburgh, Edinburgh, UK; 5 Euan MacDonald Centre for Motor Neurone Disease Research, University of Edinburgh, Edinburgh, UK; 6 Los Angeles Biomedical Research Institute, and David Geffen School of Medicine, University of California Los Angeles, Torrance, CA 90502 USA

## Abstract

Synapses are an early pathological target in many neurodegenerative diseases ranging from well-known adult onset conditions such as Alzheimer and Parkinson disease to neurodegenerative conditions of childhood such as spinal muscular atrophy (SMA) and neuronal ceroid lipofuscinosis (NCLs). However, the reasons why synapses are particularly vulnerable to such a broad range of neurodegeneration inducing stimuli remains unknown. To identify molecular modulators of synaptic stability and degeneration, we have used the *Cln3* ^−/−^ mouse model of a juvenile form of NCL. We profiled and compared the molecular composition of anatomically-distinct, differentially-affected pre-synaptic populations from the *Cln3* ^−/−^ mouse brain using proteomics followed by bioinformatic analyses. Identified protein candidates were then tested using a *Drosophila* CLN3 model to study their ability to modify the CLN3-neurodegenerative phenotype *in vivo*. We identified differential perturbations in a range of molecular cascades correlating with synaptic vulnerability, including valine catabolism and rho signalling pathways. Genetic and pharmacological targeting of key ‘hub’ proteins in such pathways was sufficient to modulate phenotypic presentation in a *Drosophila* CLN3 model. We propose that such a workflow provides a target rich method for the identification of novel disease regulators which could be applicable to the study of other conditions where appropriate models exist.

## Introduction

Synapses are an early pathological target in a range of diseases^1,2^ including conditions associated with advancing age (e.g. Alzheimer (AD)^3,4^ and Parkinson disease^5,6^), neurodevelopmental conditions (e.g. spinal muscular atrophy (SMA)^7,8^), protein misfolding/accumulation diseases (e.g. Huntington disease (HD)^9^), prion diseases^10^, spinocerebellar ataxias (SCA)^11^ and lysosomal storage disorders (Neuronal ceroid lipofuscinosis (NCLs or Batten disease)^12–16^. However, our understanding of the reasons why specific synaptic populations are so vulnerable to such a broad range of neurodegenerative stimuli, and the mechanisms that govern their stability, remains in its infancy^2,17,18^.

In this study, we sought to define the molecular regulators of synaptic stability, using animal models of CLN3 disease (a.k.a. juvenile NCL or JNCL, OMIM # 204200). The NCLs, are the most frequent autosomal-recessive neurodegenerative disease and form of dementia in childhood^19^. Incidence in the USA is estimated at 1.6–2.4/100,000 whereas in Scandinavian countries it is 2–7/100,000^20,21^. The term “NCL” currently encompasses up to 14 disease subtypes, which are grouped together due the lysosomal accumulation of autofluorescent storage material, distinct ultrastructural properties, broadly similar pathology and clinical features and a severe neurodegenerative phenotype^16,22^. Crucially for the purposes of this study, pre-synaptic disruption is a key early event in NCL, accurately predicting the distribution of subsequent neuronal loss^12–16^.

The knowledge of the underlying genetic cause and/or storage material composition have provided a base for the basic understandying of the pathogenesis and their correlation to the clinical progression of the disease, the design of gene replacement therapies and the development of animal models^23–25^. However, the pathways and molecular cascades leading to neurodegeneration in NCL, as in many other neruodegenerative diseases, are still unknown.

Mutations in *CLN3* underlie a juvenile form of NCL, the most prevalent form worldwide^26^. The *CLN3* gene encodes a putative transmembrane protein whose function is not completely understood. However, experiments in yeast and *in vitro* studies have revealed possible functions relating to vacuolar pH regulation^27^ and endocytic membrane trafficking^28^. Although *CLN3* is ubiquitously expressed throughout the body, the most obviously affected tissues are neurologic based. This feature is shared by other monogenetic neurodegenerative conditions such as SMA^29^. Yet, the reasons why neurons appear to be particularly vulnerable to defects in such broadly expressed proteins is not understood.

In humans, the onset of CLN3 disease occurs typically between 4–7 years of age, when loss of visual acuity is identified. Learning deficits are followed by speech and motor difficulties and seizures. As no treatment is currently available, premature death results at a mean age of 24 years old^30^. Unlike other more complex neurodegenerative diseases, the growing knowledge of the NCL-causative genes has facilitated the development of powerful animal models in recent years. These models have significatively improved our understanding of the progressive nature of the different forms of NCL. Cln3 null mice (*Cln3* ^−/−^) reproduce various aspects of the human disorder^31,32^. Although neuronal loss is widespread in terminal disease^31,33^, differential vulnerability can be detected across brain regions and their respective resident cell types/subcellular compartments at specific time-points. This differential degenerative progression follows the same pattern in the vast majority of NCL murine models: during pre-symptomatic stages there is an early selectivity for relay neurons within the thalamic nuclei followed by the corresponding cortical areas^14,31–35^, as well as GABAergic hippocampal interneurons and Purkinje neurons^31,32^. These reports in murine models regarding the vulnerability of the thalamus correlates with MRI studies in human patients showing alterations in thalamic areas at “pre-clinical” stages^30,36,37^. Furthermore, differential regional/neuronal vulnerability is also a shared event in a great number of neurodegenerative diseases in which some brain regions seem affected earlier than others, such as in Alzheimer^38–40^, Parkinson^41^, Huntington diseases^42,43^.

In this study, we aimed to define the molecular regulators of both synaptic stability and vulnerability using animal models of NCL. We initially characterized differential patterns of pre-synaptic pathology in *Cln3* ^−/−^ mice^31^ across two different time-points. This enabled the subsequent application of high-throughput proteomics in order to map the molecular fingerprint of differentially vulnerable biochemically-isolated pre-synaptic populations. We identify multiple cascades correlating with synaptic vulnerability and describe valine degradation and rho signalling pathways as two major regulators of synaptic vulnerability. The direct contribution of these pathways to neurodegeneration was confirmed *in vivo* using a *Drosophila* CLN3 model. Finally, we are confident that regulators of synaptic vulnerability and degeneration identified in the context of NCL are also conserved in their expression across other neurodegenerative conditions, in which pre-synaptic alterations are an early event^2^. This research therefore opens a window for further investigation into common molecular therapeutic targets and strategies for novel interventions across a range of neurodegenerative conditions during early disease onset.

## Results

### Quantitative immunohistochemistry identifies differential rates of pre-synaptic pathology between brain regions in *Cln3*^*−/−*^ mice

**Figure 1:**
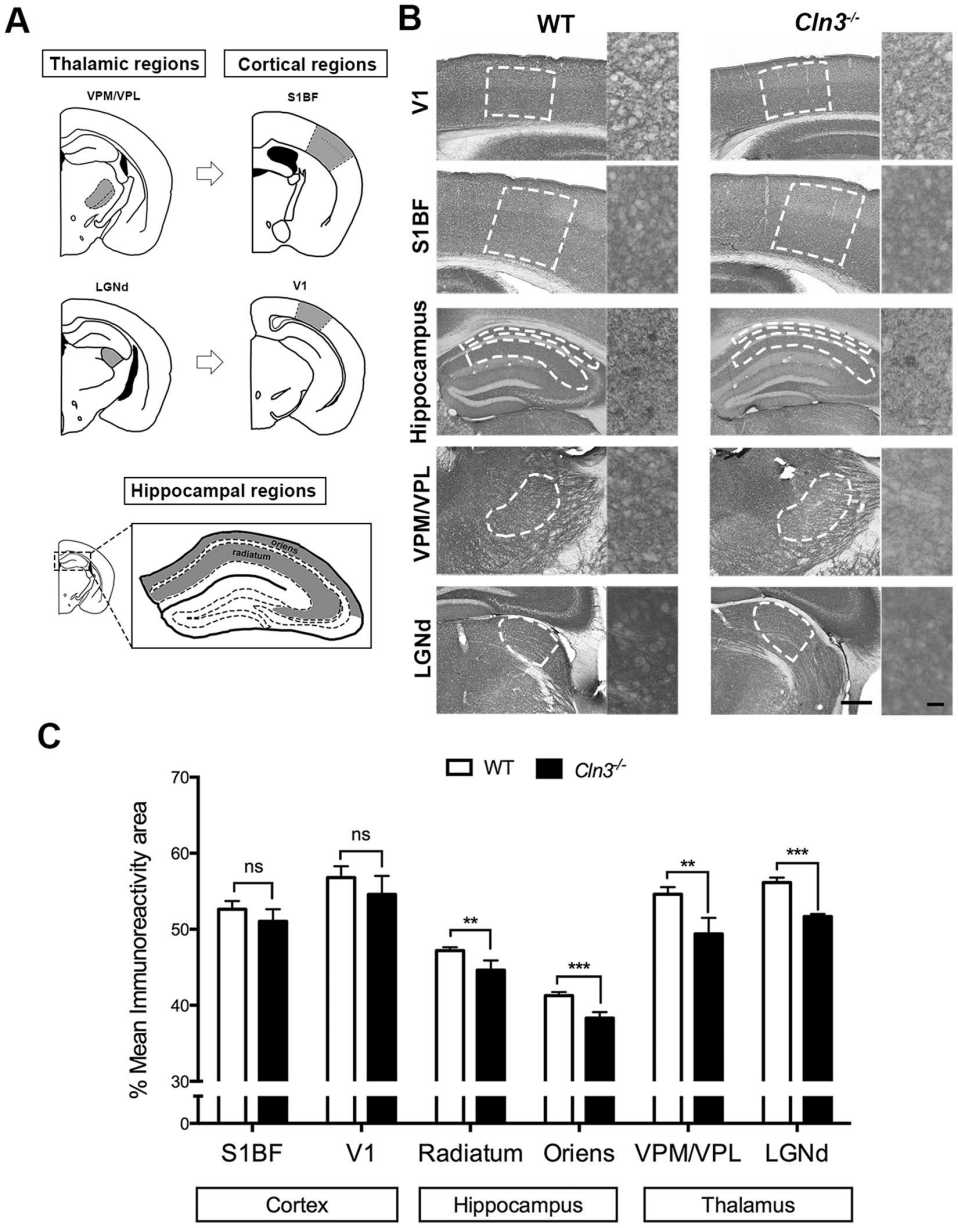
Spatio-temporal synaptic loss study in *Cln3* ^*−/−*^ detected differentially vulnerable synaptic populations across brain regions. (**A**) Brain region schematic showing the brain areas measured in grey. Thalamic regions includes the ventral posterior medial/ventral posterior lateral thalamic nucleus (VPM/VPL) (top left) and the dorsal lateral geniculate nucleus (LGNd) (bottom left); their respective cortical projections in the primary somatosensory cortex (S1BF) (top right) and primary visual cortex (V1) respectively (bottom right); hippocampal regions measured within the CA1-3 were the stratum radiatum and stratum oriens (bottom). (**B** and **C**) Representative photomicrographs of coronal sections of the same brain regions immunostained with synaptophysin (Syp) and bar chart showing its corresponding quantification based on the area of immunoreactivity in 13 month old control and *Cln3* ^−/−^ mice. Syp immunoreactivity was lower in thalamic regions (VPM/VPL and LGNd) in the *Cln3* ^−/−^ mice when compared to controls indicating more pathology, detectable earlier in the thalamus. Hippocampal stratum oriens and stratum radiatum also showed reduced Syp immunostaining, although the difference between genotypes was smaller. Cortical regions did not show difference in immunoreactivity for synaptophysin indicating that no synaptic loss is happening in these cortical areas at 13 months. (Mean ± SEM; *P < 0.05; **P < 0.01; ***P < 0.001; ns P > 0.05, Student T test, 5 mice per each genotype and time-point were used, Scale bar = 200 um (left) and 20 um (right).

Although the spatio-temporal pattern of neuron loss in *Cln3* ^−/−^ mice has already been studied^32,33^, little is known about the progression of synaptic pathology across differentially vulnerable brain regions. Studies in other NCLs using *Ppt1* ^−/−^, *Cln6* ^−/−^ or *CathD* ^−/−^ mice have revealed progressive synaptic loss starting in the thalamus and followed later in corresponding cortical areas^12,14^. Hippocampal structures seem to be also affected early, although to a lesser extent than seen in the thalamus^33^. To investigate whether a similar pattern of synaptic pathology might be present in *Cln3* ^−/−^ mice we studied the expression of the presynaptic marker synaptophysin (Syp; as previously described in^12^) in three brain regions that exhibit different degrees of neuronal vulnerability using quantitative immunohistochemistry (at 6.5 and 13 months; see methods – Fig. 1 and Supplementary Fig. S1). The three brain regions studied were: 1. Thalamus – ventral posteromedial/ventral posterolateral nuclei (VPM/VPL) and lateral geniculate nucleus (LGNd); 2. The corresponding cortical target regions in the primary somatosensory barrel field (S1BF) and primary visual areas (V1) respectively, and 3; Hippocampus- stratum radiatum and stratum oriens (Fig. 1A). At 6.5 months no significant changes in Syp expression were detected between *Cln3* ^−/−^ and control mice in any of the brain regions studied (Supplementary Fig. S1). However, by 13 months, some differences between genotypes were apparent (Fig. 1B and C). Similar to mouse models of other NCLs, thalamic nuclei demonstrated greater synaptic pathology at 13 months as indicated by the lower Syp immunoreactivity to synaptophysin in *Cln3* ^−/−^ vs. controls. Hippocampal structures were also affected, although as predicted, the difference in Syp immunoreactivity between genotypes in both hippocampal subfields was less than in thalamic nuclei. In contrast, cortical regions such as S1BF and V1 (corresponding to the thalamic nuclei VPM/VPL and LGNd) showed no statistical difference between genotypes in Syp immunoreactivity at 13 months. Immunohistochemistry using antibodies against VAMP2 and SNAP-25 (two more pre-synaptic markers) also hint at pre-synaptic alterations in the thalamus (Fig. S2–S4).

Thus, the pre-synaptic alterations seen here (as determined with the markers detailed above) appears to follow a similar pattern to that previously reported for neuronal loss in mouse models of other forms of NCL^12,14^, with the thalamus being the most affected region, followed by the hippocampus, whereas cortical measurements were still unaffected at this stage of disease. We would categorize these alterations in pre-synaptic markers as being moderate at 13 months, as the pathology as quantified here is less than 10% (9.57% relative to controls).

### Comparative molecular profiling reveals proteomic perturbations that correlate with the extent of pre-synaptic alterations

Despite advances in the characterization of the mammalian synaptic proteome, little is known about how these proteins interact and the molecular mechanisms that govern synaptic vulnerability. After confirming the synaptic vulnerability pattern across three distinct brain regions, we wanted to examine if the onset of pre-synaptic pathology correlated with distinct molecular alterations in the proteome. To address this, we examined biochemically isolated pre-synaptically enriched fractions (through the generation of “crude” synaptosomes – see methods) produced from microdissected brain regions from *Cln3* ^−/−^ mice and controls at 13 months of age where we characterise the pre-synaptic pathology as thalamus > hippocampus > cortex.

**Figure 2:**
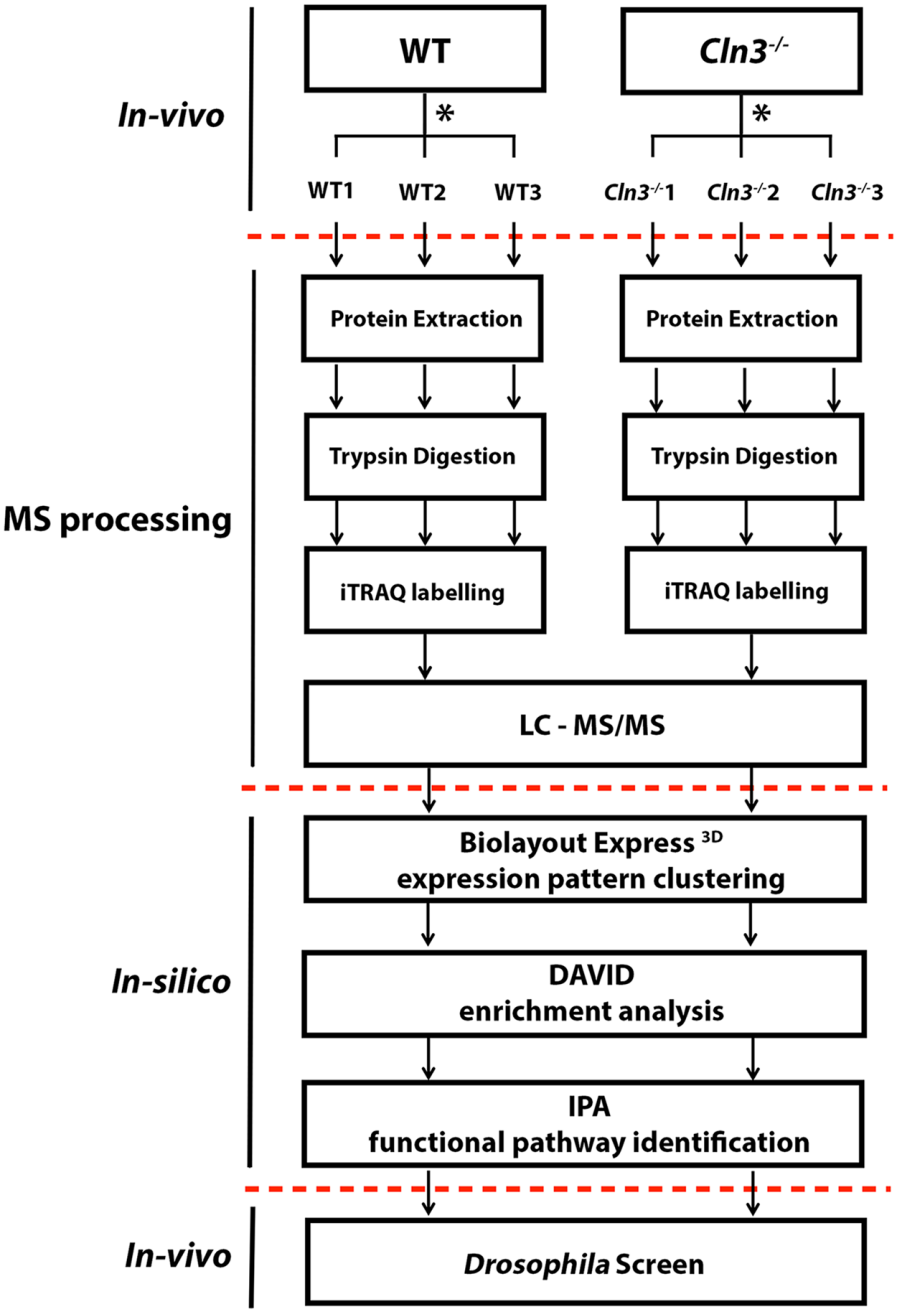
Experimental design workflow. Experiments are divided into *in vivo* (animal models), MS processing and *in-silico* where the different bioinformatics tools are detailed. *This schematic was reproduced for the three brain regions of interest: cortex, hippocampus, and thalamus at 13 months of age.

After synaptosome production and protein extraction, iTRAQ proteomics was carried out as detailed in Fig. 2. 1536 total proteins were identified in hippocampus and thalamic samples whereas 2068 were detected in cortex. We then pre-filtered the proteins by those which were identified by at least 2 peptides and therefore are more likely to be reliable identifications. 914 proteins passed that filter in hippocampus and thalamus and 1295 in cortex. Almost 40% of our pre-filtered thalamic proteome was altered when compared to control littermates. In contrast, only 20% and 17.6% of the synaptic proteome was altered in the cortex and hippocampus relative to controls respectively. The number of protein alterations also correlates with pre-synaptic pathology being more apparent in thalamic areas at this “moderate” stage of disease progression.

**Figure 3:**
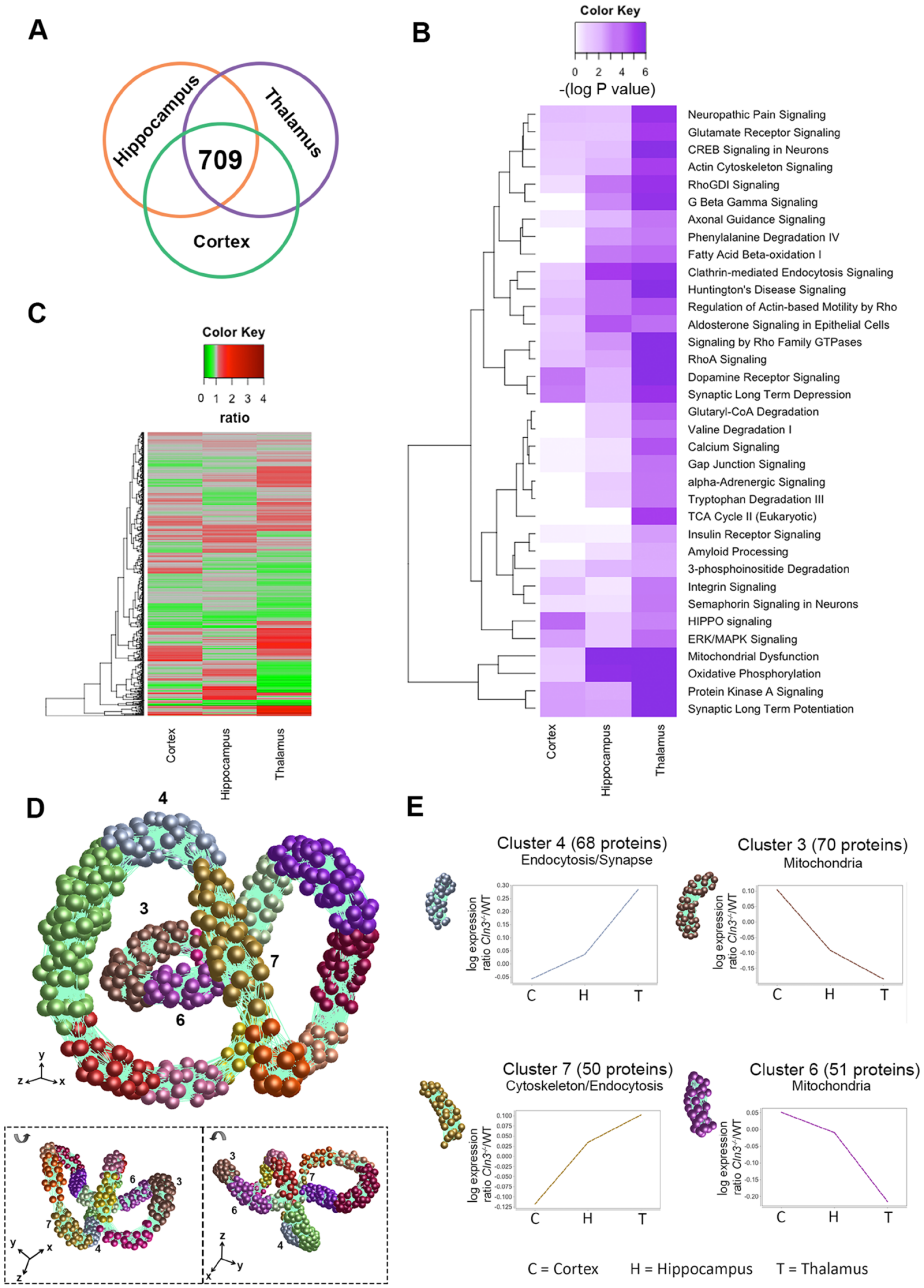
Differentially vulnerable synaptic population molecular profiling. (**a**) Venn diagram and heat map show the 709 common proteins identified and overlapped in cortical, hippocampus and thalamic synaptic proteomic datasets. (**B**) Heat map representing the significance (−log P value) of the canonical pathways identified in IPA across cortex, hippocampus and thalamus datasets. It is observed a preogressive increase in significance correlating to the synaptic vulnerability pattern described previously. (**C**) Heat map showing the 709 common proteins identified and overlapping in cortical, hippocampus and thalamic synaptic proteomic datasets. (**D**) *BioLayout* clustering 3D representation of proteomic expression data across differentially vulnerable synaptic populations orientated at 3 different angles. Each sphere represents a single protein and the edge represents how similar their expression trend is towards the other proteins in the dataset. The closer the spheres are the more similar expression trend they have. The colours represent the different clusters of co-expressed proteins. (**E**) Expression profile means in log scale (*Cln3* ^−/−^ */*WT) of co-expressed proteins in clusters 3, 4, 6 and 7 (Supplementary Tables S1) and its main biological function/subcellular compartment identified by *DAVID* enrichment analysis (See Supplementary Table S2). Clusters highlighted show steady up or downregulation across cortex (C), hippocampus (H) and thalamic (T) regions correlating with the vulnerability status of synapses.

Next, these pre-filtered proteins from the three pre-synaptic populations were aligned to look for overlapping proteins. 709 proteins were identified in all three brain regions and these were taken forward for comparative analysis (Fig. 3A).

In order to gain a broad and unbiased overview for the types of processes that may be represented by the protein alterations detected in all three pre-synaptic populations, we used *Ingenuity pathway analysis* (*IPA*) (see Methods – Fig. 3B). Interestingly, we identified multiple canonical pathways which IPA designated as perturbed in a strikingly progressive manner, consistent with the degree of pre-synaptic pathology outlined above i.e. −log P value = higher in thalamus > hippocampus > cortex (Fig. 3B). An example cascade can be found in more detail in Supplemetary Fig. S5. This analysis therefore indicates that in general, alterations in specific molecular cascades were increasing with pre-synaptic pathology, further confirming the differential vulnerability pattern previously identified by IHC. Thus, the synaptic vulnerability pattern described here appears consistent at both the morphological and that molecular level.

Whilst canonical cascade analysis is an interesting tool to use at the outset, multiple members of the same cascade do not necessarily need to change in the same manner, and whilst the trend of the majority of canonical cascades seen in Fig. 3B are consistent with the regional pathology reported, the individual protein alterations are far more complex (ass seen in the heatmap in Fig. 3C). Thus, to better understand and visualize the different molecular expression trends occurring across the three datasets, individual proteins were tracked using *BioLayout Express 3D* (see Methods). Simplistically, *BioLayout* is a complex pattern recognition software which generates a visual representation of the data based on protein abundance^44^. In this case the software interrogates a regional expression profile generated by arranging the individual proteomic sets in order of increasing pre-synaptic pathology/vulnerability (cortex < hippocampus < thalamus). The outcome of this is that proteins were found to cluster into 14 different groups according to similarities in their relative expression across these three regions (Fig. 3D)^45^. Of interest to us were Clusters 3 (70 proteins), 4 (68 proteins), 6 (51 proteins) and 7 (50 proteins) comprise proteins whose expression can be grouped (or clustered) together as having either a gradual upregulation or downregulation, therefore correlating (directly or inversely with) with pre-synaptic vulnerability (Fig. 3D and E, Supplementary Table S1). Next, we applied an enrichment analysis to these distinct clusters in order to determine if each expression trend could be associated with a specific biological or functional category (using the *DAVID* software tool – see methods and Fig. 3E). Unexpectedly, clusters with proteins that were increasingly upregulated in vulnerable regions were enriched for structural associated candidates like cytoskeletal-related proteins, and specific biological functions such as endocytosis (Cluster 4 and 7). Perhaps more surprisingly, clusters whose protein expression decreased with increasing vulnerability were related to specific mitochondrial functions (Clusters 3 and 6) (Fig. 3D and E and Supplementary Table S2). Thus, comparative proteomic profiling of differentially vulnerable pre-synaptic populations revealed that significant alterations to the synaptic proteome correlated with the degree of pathology seen at these relatively moderate disease stages.

### *In silico* analysis highlights alterations in valine catabolic and ROCK2 signalling cascades in vulnerable pre-synaptic compartments

**Figure 4:**
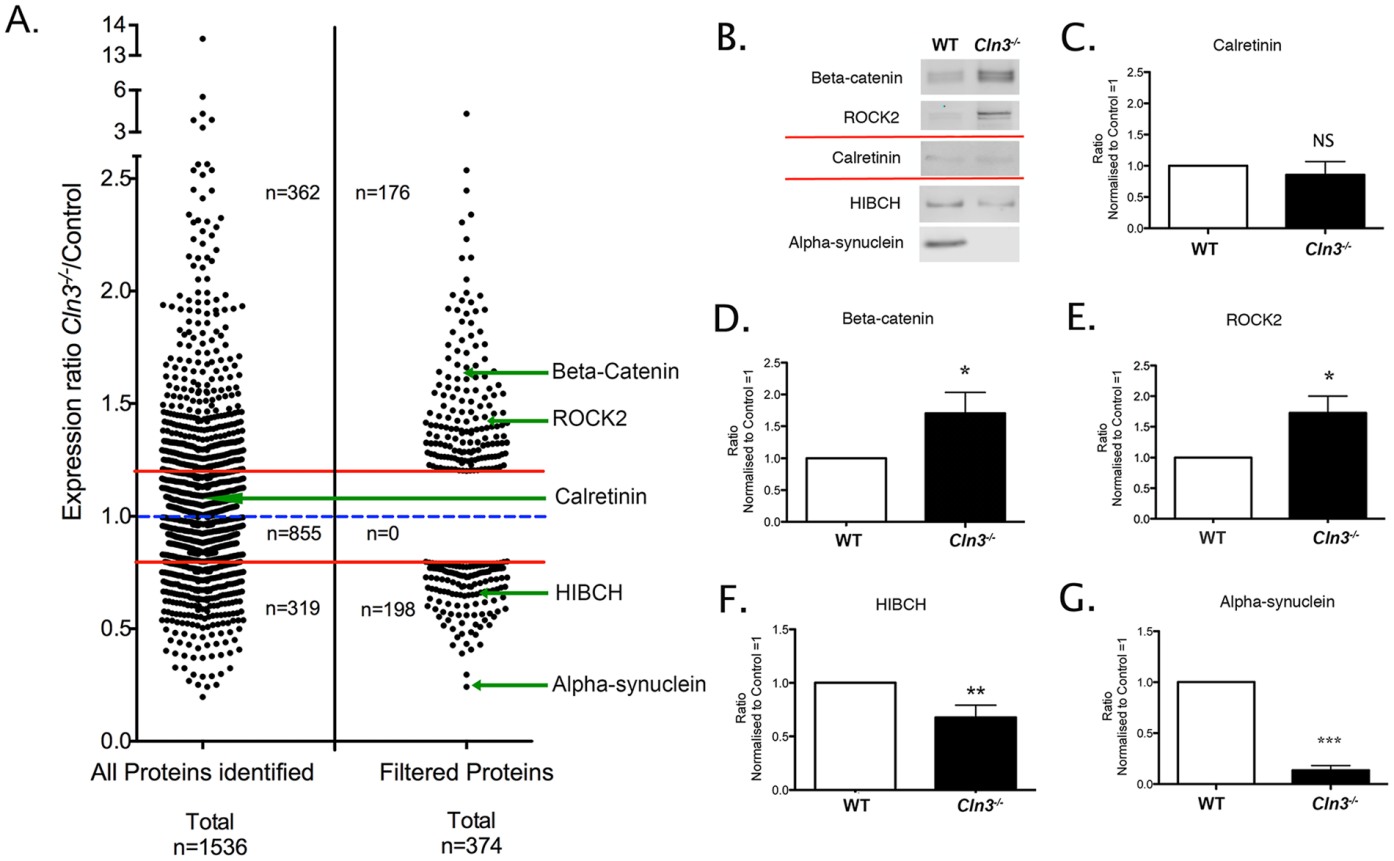
Synaptic thalamic proteome filtering and validation. (**A**) Dot plot demonstrating the process of proteomic data filtering. Each data point represents an individual protein identified using iTRAQ proteomic technique. LHS 1536 proteins were identified across all thalamic samples. Following filtering (see Methods) a molecular fingerprint for thalamic synaptic alterations comprising 374 candidate proteins was produced (RHS; see also Supplementary Tables S3 and S4). (**B**) QWB representative bands for two upregulated (β-catenin and ROCK2), two downregulated (HIBCH and α-synuclein) and one unchanged protein candidate (calretinin) verifying the proteomic data. (**C** and **G**). Quantification and statistical QWB analysis showing the magnitude of alteration in *Cln3* ^−/−^ thalamic synaptic fractions. All the selected candidate proteins were altered as indicated by this iTRAQ analysis. Mean ± SEM; *P < 0.05; ***P < 0.001 (Student T test, n = 6 mice per each genotype).

After confirming that the pre-synaptic proteome changes in a manner consistent with the differential vulnerability/regional disease progression in *Cln3* ^−/−^ mice, we next sought to tease out the potential molecular regulators of neuronal stability from our complex proteomic datasets. To do this, we focused our analyses on the thalamus, because pre-synaptic compartments here were perturbed to a greater extent than those in the hippocampus or cortex (according to the pre-synaptic markers we employed). In the thalamic dataset, 1536 total proteins were identified following iTRAQ processing. Further *DAVID* enrichment analysis was applied to the unfiltered thalamic data to confirm that the starting material was suitably enriched for synaptic proteins (Table 1). As described above, we applied filtering criteria based on the number of peptides (more than 2 unique peptides), those changed > 20% (1.2 fold-change) and those which were mapped by *Ingenuity Pathway Analysis* (IPA) software. A total of 374 proteins met these criteria (Fig. 4A and Supplementary Tables S3–S4). Next, we confirmed the veracity of the filtered data by validating a range of proteins using quantitative fluorescent Western blotting (QWB) (Fig. 4B–G).

**Table 1:** *DAVID* analysis of proteomic data confirms synaptic protein enrichment.

Term	Fold Enrichment	P-Value	Benjamini
Clathrin coat of coated pit	20.5	9.30E-02	2.70E-01
Synaptic vesicle membrane	9.7	3.70E-02	1.50E-01
Synaptic vesicle	8.2	1.30E-05	1.20E-04
Dendritic spine	6.8	7.00E-02	2.20E-01
Presynaptic membrane	6.6	7.50E-02	2.30E-01
Axon	5.2	3.40E-04	2.50E-03
Mitochondrial inner membrane	5.2	7.10E-11	1.90E-08
Synaptosome	4.7	2.20E-02	9.50E-02
Synapse	4.1	2.20E-07	4.30E-06

#### Higher order functional clustering highlights similarities with other neurodegenerative conditions

**Figure 5:**
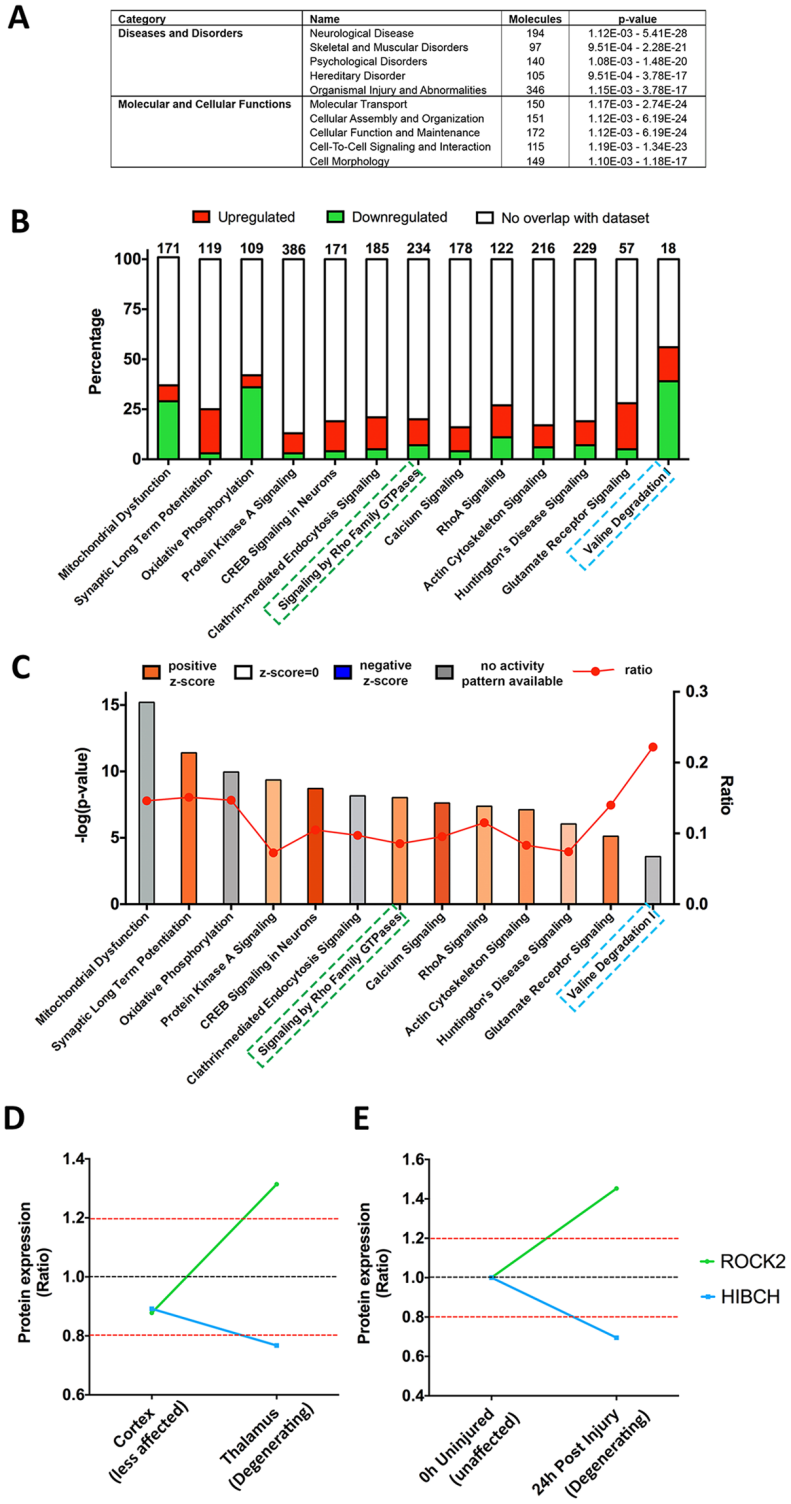
IPA analysis of the thalamic synaptic proteome. (**A**) Table of top 5 “diseases and disorders” and “molecular and cellular functions”. (**B** and **C**) Canonical pathways bar chart of representative pathways showing, (**B**) The percentage of upregulated and downregulated proteins within each pathway. Numbers on the top indicate the total number of proteins within the canonical pathway. (**C**) Significance of the association between the dataset and the canonical pathway (−log(p-value) and ratio) and z-score prediction of activation/inhibition (see Methods). Pathways highlighted in green and blue are associated to ROCK2 and HIBCH, respectively. (**D**) HIBCH and ROCK2 protein expression ratio across cortex (“less affected’) and thalamic (“degenerating”) synapses in these moderately affected *Cln3* ^−/−^ mice (*Cln3* ^−/−^/WT). (**E**) HIBCH and ROCK2 protein expression is conserved in models of injury, following similar pattern of expression 24 hours post-injury when synapses start to degenerate^2^.

Here, we used *ingenuity pathway analysis* (*IPA*) to elucidate which molecular pathways and biological networks were disrupted in “affected” thalamic pre-synaptic compartments. Here we input the total pre-filtered thalamic data into IPA and applied the 1.2 fold-change cut-off in the software. Of the three-hundred and seventy-four proteins recognised by *IPA*, 68.3% have previously been reported in the literature as being associated with neurological disease (Fig. 5A). The majority of the diseases and disorders which fall under the category of “neurological disease” are known to demonstrate synaptic pathology as an early event^1^. Interestingly, the main molecular and cellular functions identified in *IPA* were “molecular transport” and “cellular assembly and organization”. These functional categories are consistent with the comparative analysis of differentially vulnerable synaptic populations carried out above (see Fig. 3E), where clusters showing increases in protein expression from the levels detected in the comparatively spared cortical synapses through to greater levels again in the more vulnerable thalamic populations highlighted cytoskeletal protein alterations.

#### Molecular cascade tracking identified potentially conserved regulators of vulnerability

The “Canonical pathways” function in *IPA* revealed that, mitochondrial dysfunction was the most significant canonical pathway identified (see Methods). The contribution of synaptic mitochondria in neurodegeneration has been highlighted in recent years^46^, and mitochondrial abnormalities have also been related to CLN3 disease and other NCLs^47–50^, and our data is therefore consistent with these previous findings. However, in this study we wanted to consider pathways that have not previously been related to the NCLs before. Within the top canonical pathways identified with *IPA*, we also detect perturbations in ROCK signalling and valine degradation pathways (Fig. 5B and C). Examining these pathways, proteins such as ROCK2 and HIBCH were differentially expressed key “hub” components. Their expression was identified as tracking in *BioLayout* clusters 4 and 6 respectively correlating to the degree of pre-synaptic pathology in *Cln3* ^−/−^ mice (see above, Figs 3D and 5). Moreover, we have previously identified ROCK2 and HIBCH expression alterations in pre-synaptic striatal isolates following cortico-striatal lesion injury (Fig. 5E), and demonstrated their potential to alter neurodegeneration in an injury specific context *in vivo* ^2^. Collectively these findings hinted that there may be a common molecular cascade underlying pre-synaptic pathology following a range of neurodegeneration inducing insults (from injury through to genetic). ROCK2 signaling and valine degradation pathways and specifically proteins such as ROCK2 and HIBCH, could therefore represent potentially novel regulators of synaptic stability in an NCL disease context, which have not been investigated yet. So we next sought to determine if such candidates could have the ability to moderate NCL disease progression *in vivo*.

### Targeting the valine catabolic cascade is sufficient to modulate CLN3-induced neurodegeneration *in vivo*

**Figure 6:**
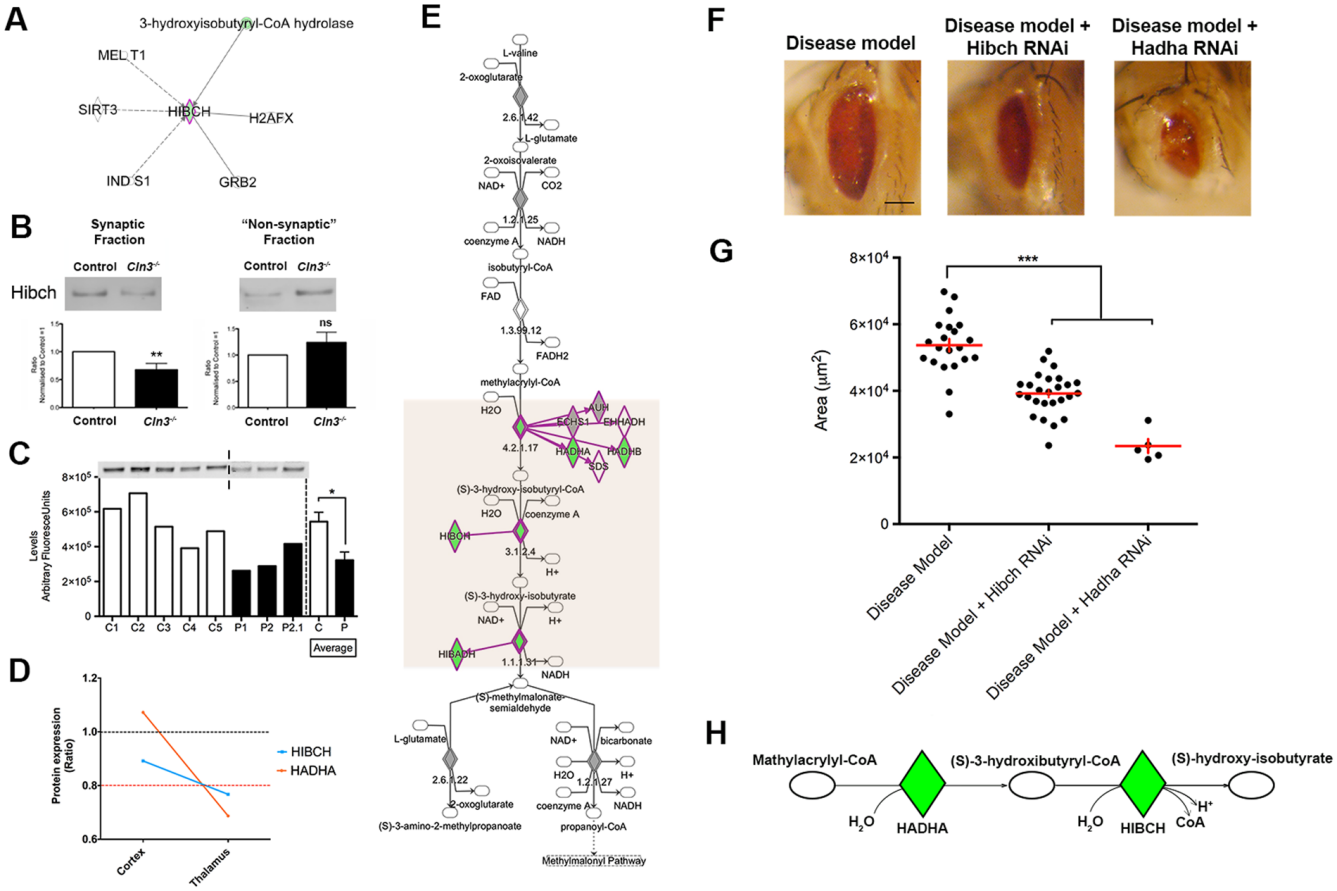
CLN3 induced degeneration is enhanced by the genetic downregulation of HIBCH and HADHA *in vivo* impacting in valine catabolism pathway. (**A**) HIBCH proteome interactome represented by IPA indicating only 6 gene/proteins are known to interact with HIBCH. (**B**) QWB bands and quantification of HIBCH in synaptic and “non synaptic” fractions of *Cln3* ^*−/−*^ and control mice in thalamic protein extracts showing a downregulation in synapses and upregulation in non-synaptic parts **P < 0.01, ns P > 0.05 (Student T test). (**C**) QWB bands and quantification of cortical “crude” synaptosomes isolated from *post mortem* human JNCL patients (P) and controls (C) showing its downregulation at end stages in human disease *P<0.05 (Student T test). (**D**) HIBCH and HADHA are co-expressed and their expression trend correlates to the vulnerability status of synapses: unchanged in cortex (“spared”) and downregulated in thalamus (“degenerating”). (**E**) Valine degradation pathway represented with *IPA*. The first protein which expression is perturbed in the degradation process of valine is HADHA; upstream of HIBCH. (**F–G**) Representative light microscope images of *Drosophila* eyes of disease model (DM), DM + HIBCH RNAi and DM + HADHA RNAi and corresponding quantification of the eye surface area. Scale bar = 100um. ***P < 0.001 (One-way ANOVA and Tukey’s multiple comparison test as a post-hoc). (**H**) Schematic representation of the fourth and fifth steps of the valine catabolism cascade showing the enzymes HADHA and HIBCH catalyzing each step respectively. (**A,E & H**) Colored nodes illustrate proteins present in the thalamic dataset in *Cln3* ^*−/−*^ with respect WT. Nodes in grey represent proteins changed <20%, down-/up-regulated proteins by >20% are represented in green and red respectively. Orange box highlights the steps that are disrupted in the valine degradation cascade. Dotted lines on representative westerns indicate cropping of bands from the same membrane.

HIBCH is a 3-hydroxyisobutyryl-CoA hydrolase protein that fulfils a core function within the valine degradation cascade^51^. Mutations in *HIBCH* cause a progressive infantile neurodegeneration in humans, characterized by hypotonia, motor delay and neurological regression^51–53^. Moreover, manipulation of HIBCH has been suggested to modulate injury-induced axonal degeneration^2^. However, despite this apparently restricted function, very little is known about its role (if any) in synaptic compartments or its binding/interaction partners (see Fig. 6A).

Using QWB techniques we confirmed that HIBCH protein was less abundant in “crude” synaptosome extracts from thalamus in *Cln3* ^−/−^ mice relative to wild type controls. Interestingly, HIBCH protein levels trended towards a slight up-regulation of HIBCH in “non-synaptic” compartments. Whilst not statistically significant, this non-synaptic increase may indicate a redistribution or altered trafficking of this protein in response to *Cln3* deficiency (Fig. 6B). Importantly, examination of human *post mortem* brain samples showed that HIBCH protein levels were significantly reduced in JNCL patients (Fig. 6C).

From comparing synaptic vulnerability (Fig. 3), HIBCH belonged to Cluster 6 (gradual downregulation profile) and was co-expressed with other mitochondria-related proteins (Fig. 3E) including Hydroxyacyl-CoA Dehydrogenase/3-Ketoacyl-CoA Thiolase/Enoyl-CoA Hydratase (Trifunctional Protein), Alpha Subunit (HADHA), which also fulfils an enzymatic role within the valine degradation pathway (Fig. 6D). HADHA sits upstream of HIBCH in the valine catabolism cascade (Fig. 6E) and catalyzes the final three steps of mitochondrial long chain fatty acid β-oxidation^54^. HADHA codes for the α subunit of the mitochondrial trifunctional protein (αMTP), and its deficiency causes a metabolic disease that presents with Reye-like syndrome, with cardiomyopathy, neuromyopathy and sudden death in infancy^55^. More recent reports have indicated that HADHA deficiency results in a pigmentary retinopathy leading to vision loss^56^.

Given the important role of these two proteins in the valine catabolism cascade, we wanted to explore the consequences of experimental suppression of HIBCH and HADHA levels *in vivo* in order to assess their influence on synaptic and neuronal stability in an NCL-disease specific context. To do so, we used a *CLN3* gain of function fly model previously characterized by Tuxworth *et al*. in which the *Drosophila* eye undergoes degeneration (becoming smaller and rougher)^57^. Although this is an accepted model for investigations into the cascades underpinning CLN3, the human disease is normally caused by loss of function. As with the human disease where dose is crucial to phenotype (i.e. heterozygous humans are not obviously affected) we can also demonstrate that degeneration ocuring in the *Drosophila* eye is CLN3 dose dependent (Fig. S6) as is the case for other neurodegeneration associated proteins (i.e. TDP43)^58^.

Thus, using this *Drosphila* model as a rapid screen for candidate ability to effect CLN3 driven phenotype (whilst bearing in mind its potential limitations) we first established a recombinant fly containing the *GMR-GAL4* eye driver and the *UAS*-*CLN3* gene in the second chromosome (*GMR-GAL4;UAS-CLN3/CyOGFP*, see Table 2), and crossed this with fly lines containing RNA interference (RNAi) of HIBCH and HADHA mouse orthologs. CG5044 and CG4389/MTPα orthologs were selected using the DRSC Integrative Ortholog Prediction Tool (DIOPT) (see Methods and Table 2). Progeny of the relevant genotype was counted (*GMR-GAL4;UAS-CLN3/HibchRNAi* and *GMR-GAL4;UAS-CLN3/MTPαRNAi*), eyes photographed and total surface area measured with *Image J* (see Methods). As expected, experimental suppression of either HIBCH or HADHA increased the neurodegenerative phenotypes observed (i.e. acting as enhancers of the CLN3-dependent phenotype) (Fig. 6F and G). Interestingly, the further upstream the valine degradation cascade is disrupted, the greater the effect on CLN3 phenotype (Fig. 6H). Interestingly, in this context neither HIBCH or HADHA were able to cause degeneration in the eye in the absence of the CLN3 disease background (Supplementary Fig. S7). This suggests that their manipulation alone is not sufficient to affect general neuronal stability, and that the effect seen when crossing HIBCH and HADHA with the CLN3 fly is likely mediated by the *CLN3* allele. As such HIBCH and HADHA appear to be *in vivo* modifiers of neuronal stability in a CLN3-disease context, and this provides further evidence that valine catabolism may play a role in CLN3-dependent neurodegeneration.

**Table 2:** *Drosophila* stock description.﻿

Line	Source		Description		
Canton S	BDSC		Wild type line		
GMR-GAL4	BDSC		Eye expression driver		
UAS-CLN3	Tuxworth *et al*. 2009		CLN3 overexpression		
GMR-GAL4;UAS-CLN3/CyO GFP	This study		Recombinant line		
**Candidate lines**	**Source**	**Genotype**	**Annotation symbol**	**Stock number**	**DIOPT score**
HibchRNAi	VDRC	w[1118]; P{GD11513}v40570	CG5044	v40570	9
MTPαRNAi	VDRC	w[1118]; P{GD11299}v21845	CG4389	v21845	8
RokRNAi GD	VDRC	w[1118]; P{GD1522}v3793	CG9774	v3793	9
RokRNAi TRiP	BDSC	y[1] v[1]; P{y[ + t7.7] v[ + t1.8] = TRiP.JF03225}attP2	CG9774	28797	9

VDRC: Vienna *Drosophila* RNAi Centre.

BDSC: Bloomington *Drosophila* Stock center.

### Increased pre-synaptic ROCK2 expression appears conserved across a range of diseases and its downregulation can reduce CLN3-induced neurodegeneration *in vivo*

**Figure 7:**
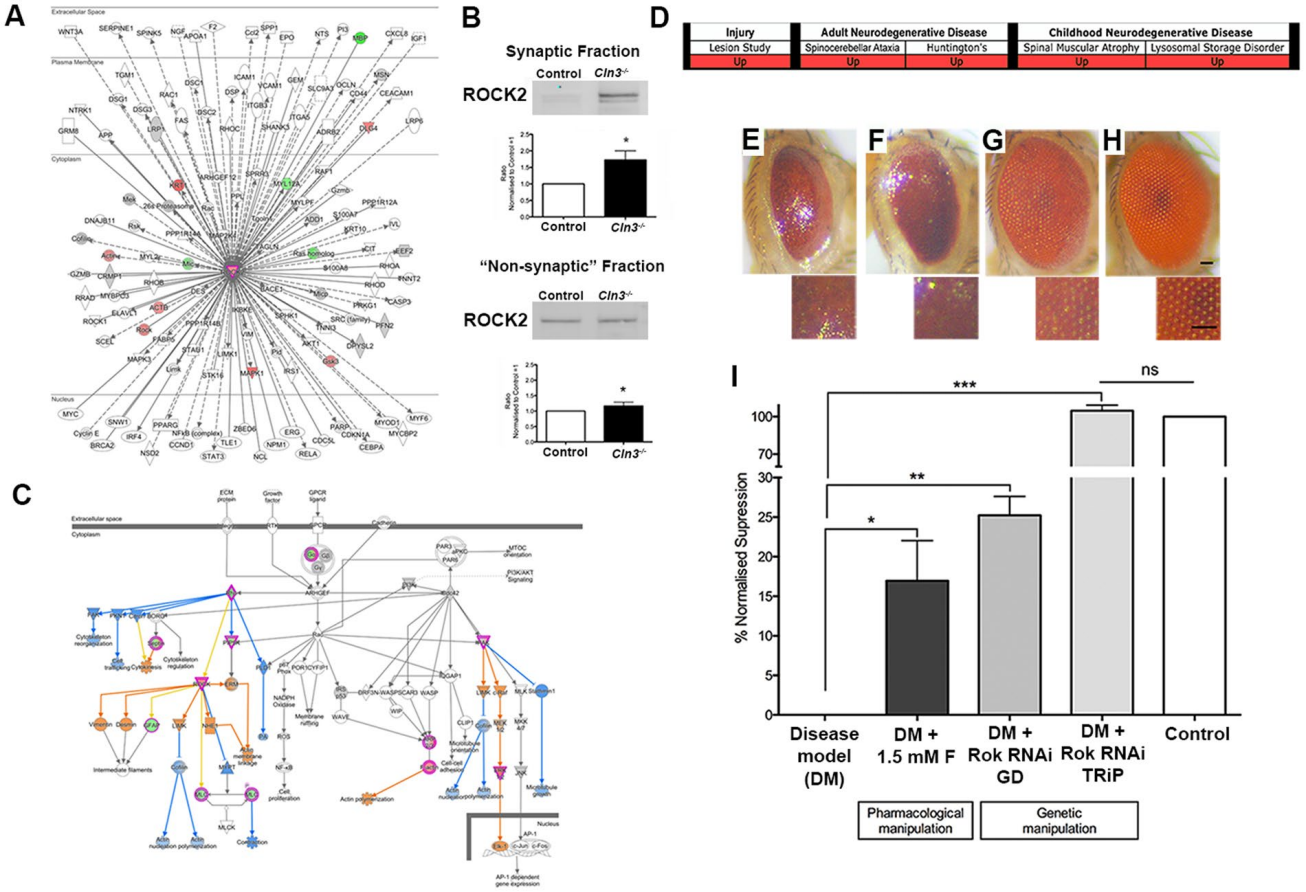
CLN3 induced degeneration is suppressed by the downregulation of ROCK2 genetically and pharmacologically (**A**) ROCK2 protein/gene interactome represented with IPA showing that some of the interactors of ROCK2 are also altered in “crude” thalamic synaptosomes. (**B**) QWB bands and quantification of control and *Cln3* ^−/−^ synaptic and “non synaptic” fraction. Upregulation of ROCK2 is higher in synapses than in the non-synaptic fraction indicating a potential synaptic specific response. *P < 0.05 (T-student test). (**C**) “Signaling by Rho family GTPases” canonical pathway represented by *IPA*. Upregulation/activation of ROCK2 impacts in actin nucleation and polymerization that may disrupt actin dynamics in the synapse. (**A&C**) Coloured nodes illustrate proteins present in the thalamic dataset in *Cln3* ^−/−^ with respect WT. Nodes in grey represent proteins changed <20%, down-/up-regulated proteins by >20% are represented in green and red respectively, orange indicates predicted activation and blue; predicted inhibition. (**D**) ROCK2 upregulation at early stage of disease is a converved event across animal models of injury, two adult-onset neurodegenerative diseases (Huntington and Spinocerebelar ataxia)^2^ and two childhood neurodegenerative conditions (SMA and the lysosomal storage disorders-NCLs). (**E** and **H**) Representative light microscope images of *Drosophila* eyes and corresponding 200x zoom of eye structure of (**A**) Disease Model (DM) (**B**) DM + 1.5 mM Fasudil. (**C**) DM + Rok RNAi TRiP and (**D**) Control fly (Canton S). (**I**) Bar chart representing the % of normalized suppression calculated from the average eye surface areas of three independent experiments as “(x- average eye surface area of the disease model)/(average of eye surface area of the control - average eye surface area of disease model) × 100” for each of the three independent experiments. Scale bar = 100um. ***P < 0.001, *P < 0.05 (One-way ANOVA and Tukey’s multiple comparison test as a post-hoc).

As discussed above, ROCK cascades were also identified as being perturbed in degenerating thalamic pre-synaptic compartments and were predicted (by *in silico* analysis) to be activated when compared to wildtype controls (Fig. 5B and C). ROCK2 is a *Rho*-kinase belonging to a family of serine/threonine kinases, of which isoform 2 is the most predominant in the brain^59^. ROCK2 directly interacts with, and is activated by, Rho GTPases. These are the central mediators of actin reorganization^59^, which are reported to have a key role in synaptic plasticity and long term potentiation^60^. Activation of ROCK2 has been implicated in several adult-onset neurodegenerative conditions where synaptic pathology is present, such as HD, AD, ataxia, and in Purkinje cell degeneration^2,61–64^. Moreover, ROCK2 has been shown to influence childhood neurodegenerative conditions such as SMA, where pharmacological inhibition partially recued symptoms and increases lifespan in an SMA mouse model^65,66^. Moreover, we have previously reported that ROCK2 is capable of influencing axonal degeneration *in vivo* following injury^2^. Taken together with our current data, this is consistent with a model whereby upregulation/activation of ROCK2 signalling in pre-synaptic compartments may correspond with increased neuronal vulnerability or degeneration in multiple infantile- and late-onset neurodegenerative conditions (Fig. 7D).

The ROCK2 interactome (displayed in Fig. 7A) illustrates its reported interactions with other gene/proteins, many of which were also altered in *Cln3* ^−/−^ thalamic synaptic extracts. In the *Cln3* ^−/−^ thalamus, QWB confirmed that ROCK2 was upregulated by 53.7% in the synaptic fractions, and by 27.7% in the corresponding “non synaptic” *Cln3* ^−/−^ isolates, relative to controls (Fig. 7B), suggesting a more synaptically-focussed response to altered CLN3 expression. Our *in silico* analysis added support for this model by highlighting specific sub-cascades under multiple pathways related to ROCK2 such as “Signalling by Rho Family GTPases” and “Rho A signalling” (Fig. 5B and C). These cascades regulate actin dynamics and cytoskeletal organization (as shown in Fig. 7C) and can impact synaptic structure and affect synaptic transmission. Figure 7D summarises previously published data generated by our laboratory where ROCK2 was identified to be upregulated in a range of conditions at early stages of disease progression^2^ and new synaptic data regarding its role in CLN3 disease. Thus, the conserved increased expression of ROCK2 in *Cln3* ^−/−^ mice, and in other related conditions and its ability to change degeneration, is highly suggestive of a potentially conserved contribution to synaptic vulnerability.

Thus, in order to investigate whether ROCK2 is capable of directly modulating the process of neurodegeneration in a CLN3-specific disease context *in vivo*, we again turned to the CLN3 *Drosophila* model. RNAi GD and RNAi TRiP fly lines for a ROCK2 ortholog were crossed to our established CLN3 recombinant *Drosophila* (see Table 2). Genetic downregulation of ROCK2 with two independently generated RNAi lines were tested and both were found to drastically improve their CLN3*-*induced small eye phenotype, although was more obvious with the TRiP library line (Fig. 7G–I). The total surface area of the eyes of the offspring (*GMR-GAL4;UAS-CLN3/RokRNAi*) were non-significant with respect the control eyes and therefore the phenotype was fully-recued. Qualitatively, there was also an obvious reduction/absence of black patches (indicative of cell death), and clear recovery of the gross structure of the *Drosophila* compound eye. With the RNAi GD line the eye size was also improved (25.22% of recue) (Fig. 7I). Thus, experimental manipulation of ROCK2 was sufficient to modulate neurodegeneration in a CLN3 model *in vivo*. To exclude the possibility of the suppression effect being due to the dilution of *GAL4* among two UAS transgenes, a control experiment was carried out, where the CLN3 recombinant fly was crossed to a UAS-*GFP* line. No significant changes were detected in the eye of the offspring, indicating that the results showed in our RNAi experiments are likely a consequence of the downregulation of ROCK2.

### The ROCK inhibitor Fasudil ameliorates CLN3-dependent neurodegeneration *in vivo*

Following the rescue of the CLN3-dependent eye degeneration by means of genetic tools we therefore wanted to test the possibility of targeting ROCK2 pharmacologically in *Drosophila*.

Several ROCK inhibitors have been characterized, but their specificity for individual ROCK kinases is still unclear. Fasudil is a small molecule that unspecifically inhibits ROCK2, originally discovered as a blocker of cerebral vasospasm in animal models^67^. Importantly, fasudil has been demonstrated to be safe and effective in clinical trials of cerebral vasospasm, pulmonary hypertension, Raynaud phenomenon and cancer^68–73^. Moreover, fasudil has previously been applied to murine models of SMA showing an improvement of the phenotype and lifespan of these mice^65,66^. However, fasudil also targets other kinases such as MAP4K4, PKC, PRKAA1 or PRKAA2^74^, which are predicted to be also activated by IPA (Supplementary Fig. S8A). The activation of these kinases seem to have downstream consequences on biological functions relevant to NCL such as autophagy (Supplementary Fig. S8A) or and “Protein kinase A signalling” (Supplementary Fig. S8B and C). Given the success of the compound in mammalian systems^65,66,73^, including humans^69,70^, to date and the apparently conserved ROCK2 alterations across multiple-neurodegenerative paradigms, we decided to test Fasudil in CLN3 *Drosophila* (*GMR-GAL4/UAS-CLN3*). The drug was added into the fly food in order to obtain 1.5 mM of fasudil in the food and the *Drosophila* were raised as normal (see Methods). Pharmacological inhibition with fasudil caused a significant, albeit modest suppression of the CLN3*-*derived eye phenotype. Quantification of eye surface areas confirmed a 17% rescue of the CLN3 eye phenotype in the treated animals (Fig. 7E,F and I). Thus, targeting of ROCK cascades with fasudil was sufficient to ameliorate neurodegeneration in a CLN3 model *in vivo*.

## Discussion

Here we show what may be the first proteomic profiling carried out across multiple differentially vulnerable synaptic populations at an early stage of disease progression. We have demonstrated that such unbiased proteomic mapping of distinct pre-synaptic populations coupled with *in silico* analysis, and *in vivo* rapid phenotypic screening in *Drosophila*, is an effective target-rich workflow for the identification of novel molecular alterations that regulate synaptic/neuronal stability.

Here we have confirmed that *Cln3* ^−/−^ murine pre-synaptic pathology appears to follow the same neuronal differential vulnerability pattern as other NCL subtypes^12,14^. We show that, at 13 months of age in *Cln3* ^−/−^ mice the synaptic pathology is more pronounced in the thalamus (although still <10%), followed by hippocampal pre-synaptic compartments, while cortical synaptic populations remain relatively unaffected (Fig. 1). iTRAQ proteomic and bioinformatics showed that differential synaptic pathology was also reflected at the molecular level, showing a progressive increase in the disruption of multiple canonical pathways correlating with our observations at the immunohistological level (Fig. 3B). Further analyses by means of complex pattern recognition software identified alterations in the pre-synaptic proteome (identified by iTRAQ analysis of “crude” synaptosomes) that correlated with the assigned vulnerability status of pre-synaptic populations in the *Cln3* ^−/−^ mice. Pathway analysis highlighted perturbations in valine catabolism and *rho* signaling pathways, with proteins including HIBCH, HADHA and ROCK2 as key players, correlating with synaptic vulnerability. Furthermore, HIBCH and ROCK2 displayed a conserved direction of change in multiple neurodegenerative conditions (Figs 5E and 7D), and we have previously demonstrated that they can alter axonal degeneration in an injury dependent context^2^. Importantly, this suggests that not only do conserved pathways regulating degenerative processes exist, but they may be targetable across multiple conditions (Fig. 7D).

In contrast, no lysosomal specific pathways were identified in our *in silico* analysis in synapses, indicating that loss of CLN3 may be impacting in other pathways beyond lysosomal function. This is supported by studies in which CLN3 was found to be expressed in other locations such as in synaptosomes, lipid rafts, Golgi, mitochondria, glia and endothelial cells^75–79^, where it may play other roles such as regulation of oxidative stress^78,80^ and glial function^32,34^ or blood-brain barrier homeostasis^79^. However, individual NCL and lysosomal-storage disorder-related proteins were identified, such as cathepsin D^81^, prosaposin^82,83^ and acid ceramidase^84^ confirming the veractity of the data generated.

By influencing the valine catabolic cascade through genetic disruption of HIBCH and HADHA it was possible to alter the CLN3 degenerative phenotype in *Drosophila in vivo*. Moreover, the further upstream in the catabolic cascade we intervened, the greater the resulting effect on disease phenotype. In humans, both HIBCH and HADHA deficiency cause infantile onset diseases with neurologic clinical scheme^53,85,86^. Crucially, this could indicate that perturbations in valine catabolic pathways might be a shared event across multiple neurodegenerative conditions and therefore targets identified here may be transferable to other diseases.

Interestingly, the upregulation of ROCK2 has been reported in a range of conditions by our laboratory and others^2,61,62,64^ (Fig. 7D). ROCK2 upregulation seemed to be a more synaptic-specific perturbation (at least at early stages) in the *Cln3* ^−/−^ mice and its magnitude of change tracked across differentially vulnerable brain regions (i.e. Thalamus > Hippocampus > Cortex). Its genetic and/or pharmacological downregulation has also been shown to ameliorate the phenotype of several conditions including SMA, another relatively early onset neurodegenerative disease^65,66^. In this study we demonstrate for the first time that downregulation of ROCK2 also recues the phenotype in a CLN3 *in vivo* model when manipulated genetically, and to a lesser degree with available pharmacological agents (Fig. 7E–I).

Cortical synaptic extracts from human patients have shown that ROCK2 was downregulated in JNCL patients with respect to controls (Supplementary Fig. S9). Nevertheless, the interpretation of the results from *post mortem* tissue is challenging as at end stage of the disease brain atrophy is much more pronounced, making it difficult to distinguish which molecular alterations are more likely to be causative, and which are a consequence of the ongoing degeneration taking place. ROCK2 upregulation could be a disease stage-specific change that contributes to the initiation of degenerative stimuli, and it is not necessarily globally upregulated throughout disease progression. It would be advantageous to study the molecular alterations and pathways taking place throughout the time-course of disease progression in NCL as this would further our understanding of the mechanisms underlying the initiation and progression of neurodegeneration. In general, ROCK2 appears to be an attractive target for the development of new therapeutic strategies, not only for NCL, but also in a wide range of more common neurodegenerative diseases to potentially halt or delay disease progression (Fig. 7D). There are currently multiple ROCK inhibitors with different degrees of specificity available^68,74^. However, in order to avoid off-target effects in future studies, it will be necessary to develop a compound that targets ROCK2 more specifically.

It would be of interest for future investigations to study the molecular overlaps between valine degradation and ROCK2 signaling pathways contributing to synaptic vulnerability. *In silico* analysis based on published literature, suggests potential overlaps, although the level of complexity in the published data allowing us to draw links between these candidates would require carefully planned experiments in order to unravel such interactions (See Fig. S10).

We suggest that common mechanisms regulating the process of neurodegeneration and/or vulnerability of synapses may occur across a range of neurodegenerative conditions triggered by different insults. Unlike more common adult-onset complex polygenetic diseases such as Alzheimer or Parkinson disease for which the genetic insult triggering the neurodegeneration is not well understood, the NCLs are monogenetic diseases for which there are available well-characterized mice models that more accurately replicate the human disease^14,31,32,35,87,88^. Here we demonstrate that we can use the NCLs as a model to study a specific neurodegenerative process (i.e. synaptic loss) which is likely goverened by mechanisms which may indeed be conserved across multiple conditions ranging from injury through to chronic neurodegenerative conditions (i.e. Huntington disease and spinocerebellar ataxia) and diseases of childhood (i.e. SMA and the NCLs).

The workflows highlighted here should not be viewed as a mass screening “stamp collecting” exercise. Instead it should be clear that combining mammalian model “–omic” screening of differentially vulnerable tissues with *in silico* candidate identification and *in vivo* phenotypic assessment in *Drosophila* is an efficient pipeline for elucidating the mechanistic cascades governing neurodegenerative processes, and a “target-rich” way of identifying factors which are capable of modulating neurodegeneration, and therefore may be therapeutically targetable.

## Materials and Methods

### Tissue harvesting and processing

#### Ethics statement

All animal experiments were approved by a University of Edinburgh internal ethics committee and were performed under license by the UK Home Office (project license numbers 70/6567 and 70/7364).

#### Mice

*Cln3* ^−/−^ mice^31^ inbred on a C57BL/6 background for at least 10 generations and control (+/+) littermates resulting from heterozygous crosses were used in this study. Mice were genotyped as described in^31^. Five *Cln3* ^−/−^ and five wild-type control mice (WT) at 6.5 months and 12–13 months of age were used for immunohistochemistry experiment. Four *Cln3* ^−/−^ and four control mice (WT) at 12–13 months were used for the proteomics and biochemical experiments.

#### Human post mortem samples

Human brain samples were obtained from the Human Brain and Spinal Fluid Resource Center, Los Angeles and from The MRC London Neurodegenerative Disease Brain Bank, Institute of Psychiatry, Psychology & Neuroscience, King’s College London. Samples were obtained at routine autopsy with fully informed written consent by the families. Study protocols for the use of this human material were approved by the Ethical Research Committees of the Institute of Psychiatry under the approval numbers 223/00 and 181/02. All experiments using human tissue samples and/or derivatives of, were performed in accordance with the relevant guidelines and regulations governing their storage, handling and disposal. Details about the samples are described in Supplementary Table S5.

### Immunohistochemistry (IHC)

Five *Cln3* ^−/−^ and five wild-type control mice (WT) at 6.5 months and 12-13 months of age were terminated and brains rapidly removed and immersed for fixation in 4% paraformaldhyde (pH 7.4). The IHC protocol used allows quantitative and qualitative comparisons between animal tissues as previously described in^89,90^. Please refer to Supplemental Experimental Procedures for more details.

### Biochemical separation of neuronal compartments

Brains were harvested from six *Cln3* ^−/−^ and wild-type (WT) controls mice at 13 months old and briefly chilled in ice cold ACSF (125 mM NaCl, 26 mM NaHCO3, 25 mM glucose, 2.5 mM KCl, 1.25 mM NaH2PO4, 1 mM CaCl2, 4 mM MgCl2) before regional microdissection. Thalamus, cortex (predominantly pre-/frontal) and hippocampus and were microdissected bilaterally^12,17,91^ pooled by mouse and immediately processed for “crude” synaptosome production as previously described^2,17,92^. Please refer to Supplemental Experimental Procedures for more details.

For the *post mortem* human samples, a portion of the total sample was obtained from frozen tissue (stored at −80 °C) and transferred in an eppendorf tube containing cold isotonic sucrose solution. Please refer to Supplemental Experimental Procedures for more details.

### Protein extraction

#### Mice

For iTRAQ proteomics, protein extraction from “crude” synaptosomes was carried out as previously described in Fuller *et al.* ^93^. Please refer to Supplemental Experimental Procedures for more details.

#### Human

“crude” synaptosomes were resuspended and homogenized in a solution containing 100 mM Tris-HCl, 4% (w/v) SDS and 1% protease inhibitor cocktail (Roche). Samples were spun at 20,000 g and 4 °C for 20 minutes. Supernatant was aspirated and collected as extracted sample. Pellets and extracted samples were stored at −80 °C for QWB. Extracted samples concentration was determined using a BCA assay (Thermo).

### Proteomic processing

Sample preparation and protein identification and quantification analysis by mass spectrometry was carried out as previously described in Fuller *et al.* ^93^. Please refer to Supplemental Experimental Procedures for more details.

### Quantitative fluorescent western blotting (QWB)

QWB was performed as previously described in refs^2,91,92,94,95^. Briefly, samples were denatured in NuPage® LDS Sample buffer 4X (Invitrogen, UK) at 98 °C and 15ug of protein loaded and run on 4–12% Bis-Tris gel (Invitrogen). Accuracy of loading and protein estimation was confirmed by total protein analysis of Instant Blue (Expedeon) stained gels as previously described^94^. Protein transfer to a polyvinylidene fluoride (PDVF) membrane was carried out using the I-Blot® transfer system (Invitrogen, UK). Membranes were incubated with Odyssey blocking buffer (Li-Cor) for 30 minutes. Next, membranes were incubated in primary antibodies overnight at 4 °C and secondary antibodies for 1 h at room temperature. Please refer to Supplemental Experimental Procedures for more details.

### *In silico* proteomic analysis

#### BioLayout Express 3D

BioLayout software incorporates a complex pattern recognition algorithm which groups protein data based only on expression profile. It allows the visualization and graphing of expression trends of co-expressed proteins to allow better characterization and understanding of complex large datasets^44^. Please refer to Supplemental Experimental Procedures for more details.

#### Enrichment analysis

To obtain an indication of the level of sample enrichment afforded through the process of synaptosome production, un-filtered mass spectrometry data was processed using The *Database for Annotation, Visualization and Integrated Discovery* (*DAVID*) software (available at http://david.abcc.ncifcrf.gov). *DAVID* provides a relatively comprehensive set of functional annotation tools for large data set interpretation^96,97^. *DAVID* was also used to characterize the functions associated to the protein clusters correlating to vulnerability mapped using *BioLayout*. For analysis to confirm synaptic protein enrichment we have applied a cut off of four fold as previously described in^98^ (see Table 1).

#### *Ingenuity Pathway analysis*

To obtain further insight into potential cellular pathways that may be perturbed in the *Cln3* ^−/−^ thalamic pre-synaptic compartments compared to control mice, the *Ingenuity Pathways Analysis* (*IPA*) application (Ingenuity Systems) was used, as previously described^2,91,92,99^. Please refer to Supplemental Experimental Procedures for more details.

### Phenotypic assessment methods and *Drosophila* husbandry

*Drosophila* mouse orthologs for candidate proteins were identified using *DRSC Integrative Ortholog Prediction Tool* (*DIOPT*)^100^ and RNAi lines were chosen from Flybase and obtained from Vienna *Drosophila* stock centre (VDSC) and from Bloomington *Drosophila* Stock Centre (BDSC) (see Table 2). UAS-*CLN3* mutant fly was donated by Richard Tuxworth^57^. A stock carrying *GMR-GAL4* and UAS-*CLN3* on the second chromosome was established by conventional recombination methods and used as a tester line for the screen (*GMR-GAL4;UAS-CLN3/CyO GFP*). RNAi candidate lines were crossed to this line and the F1 progeny was assessed for suppression or enhancement of the CLN3-derived small and rough eye phenotype. To have a quantitative read out of the severity of the phenotypes obtained in our experiments we measured the eye surface area, as previously described^101^. Stocks were maintained on standard cornmeal food at room temperature. For all the crosses, flies were raised at 25 **°**C in a circadiarian light incubator. Details of the lines used can be found in Table 2.

### Fasudil drug assay

The rock inhibitor used was Fasudil, Monohydrochloride Salt (LC laboratories, *F-4660*), which was previously used in mice models of SMA improving survival and skeletal muscle development^65^. *GMR-GAL4* x UAS-*CLN3* crosses were raised in Nutri-Fly instant food (Genesee Scientific, *66-118*) containing 1.5 mM of Fasudil Monohydrochloride Salt and blue food colouring diluted in water, as described in^102^. Eyes of the offspring were photographed and total surface area was measured with *Image J*. Suppression of the CLN3-degenerative eye were calculated from 3 independent experiments as “(x- average eye surface area of the disease model)/(average of eye surface area of the control − average eye surface area of disease model) × 100” for each experiment.

### Imaging

#### Immunohistochemistry

Micrographs were taken with Leica DMRB × 5/0.12 objective and AxioCam HRC Zeiss from the following brain regions: 1. The thalamic nuclei ventral posterior medial/ventral posterior lateral thalamic nucleus (VPM/VPL) and the dorsal lateral geniculate nucleus (LGNd); 2. Their respective cortical projections in the primary somatosensory cortex (S1BF) and primary visual cortex (V1) respectively, and 3; the hippocampal stratum radiatum and stratum oriens (See Fig. 1A). Immunoreactivity measurements were carried out using a thresholding image analysis in *Image J* (National Institute of Health, Bethesda, MD), USA), as previously described^32,34,103^. Thirty non-overlapping images (x40) were taken, on three consecutive sections, through the brain areas described above. All parameters including lamp intensity, video camera setup and calibration were kept constant throughout image capturing.

#### *Drosophila* eye images

*For the HIBCH and HADHA experiments* fruit fly eye images were photographed with a Nikon D5100 camera attached to a SZX9 Nikon stereomicroscope. For the ROCK2 experiments eye images were taken with an AxioCam ERc 5 s Rev.2 attached to a Zeiss Stemi 305 trino stereomicroscope. Images were analyzed with *Image J* software (National Institute of Health, Bethesda, MD, USA) as previously described in^101^.

### Data analysis and figure production

QWB data was analysed using *Odyssey* software as per manufactures guidelines and as previously described^2,17,104^. Data was graphed and statistical comparisons for IHC thresholding, QWB protein intensity and *Drosophila* eye surface area quantification were carried out using *GraphPad Prizm* as previously described^91,101^. For QWB and IHC Student’s T test was applied. For *Drosophila* eye quantifications one-way ANOVA was carried out followed by Tukey’s multiple comparison test as a post-hoc when a significant difference was found in the ANOVA. P values < 0.05 were considered to be statistically significant for all analyses (*P < 0.05; **P < 0.01; ***P < 0.001). Statistical analysis of proteomic data was automatically carried out by *MASCOT*. P & Benjamini values to determine enrichment were automatically calculated by *DAVID* software (see above and http://david.abcc.ncifcrf.gov). P values/Fishers exact tests for pathway and networking analysis were automatically determined by *IPA* (see above and http://www.ingenuity.com/). Heatmap representing the differentially expressed proteins and pathways across brain regions was produced using “R” (http://www.R-project.org)^105^.

### Availability of data and material

The raw datasets used and/or analysed during the current study are available from the corresponding author on reasonable request.

## Electronic supplementary material

Supplementary Information file

Supplementary Dataset 1
