# Supplementary material for: Proteomic mapping of differentially vulnerable pre-synaptic populations identifies regulators of neuronal stability in vivo
Source: Sci Rep. 2017 Sep 29;7:12412. doi: 10.1038/s41598-017-12603-0 (PMC5622084; doi:10.1038/s41598-017-12603-0)

<u>Supplementary Information Document for:</u>

Proteomic mapping of differentially vulnerable pre-synaptic populations identifies regulators of neuronal stability *in vivo*.

Maica Llavero Hurtado[1], Heidi R. Fuller[2+], Andrew M.S. Wong[3+], Samantha L. Eaton[1], Thomas H. Gillingwater[4, 5], Giuseppa Pennetta[4, 5], Jonathan D. Cooper [3,6†] & Thomas M. Wishart [1,5†]*

<u>Contents:</u>

P2-14: Supplementary figures
P15-22: Supplementary methods and materials
P23-26: Raw western blot membrane images from main figures

Proteomic mapping of differentially vulnerable pre-synaptic populations identifies regulators of neuronal stability *in vivo*.

Maica Llavero Hurtado[1], Heidi R. Fuller[2+], Andrew M.S. Wong[3+], Samantha L. Eaton[1], Thomas H. Gillingwater[4, 5], Giuseppa Pennetta[4, 5], Jonathan D. Cooper [3,6†] & Thomas M. Wishart [1,5†]*

Supplementary Figures

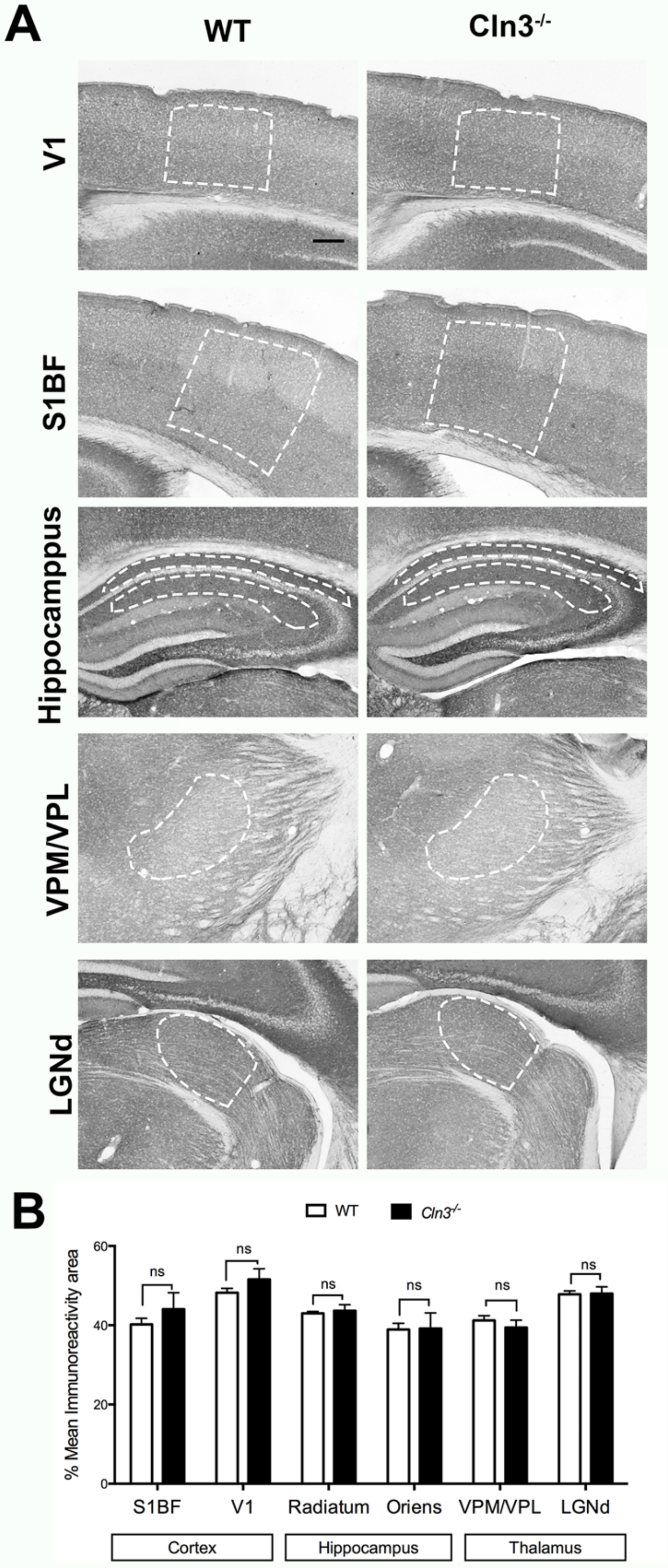
A
WT
Cln3-/-
V1
S1BF
Hippocampus
VPM/VPL
LGNd
B
WT
Cln3-/-
% Mean Immunoreactivity area
60
40
20
0
ns
S1BF
V1
Radiatum
Oriens
VPM/VPL
LGNd
Cortex
Hippocampus
Thalamus

***Fig S1.* The pre-synaptic marker synaptophysin is not significantly altered between control and *Cln3*$^{-/-}$ mice at 6.5 months:** (**a**) Representative photomicrographs of coronal sections of thalamic regions includes the ventral posterior medial/ventral posterior lateral thalamic nucleus (VPM/VPL) and the dorsal lateral geniculate nucleus (LGNd); their respective cortical projections in the primary somatosensory cortex (S1BF) and primary visual cortex (V1) respectively; hippocampal stratum radiatum and stratum oriens from CA1-3 (bottom) immunostained with synaptophysin and a histogram showing its corresponding quantification based on Syp immunoreactivity a at 6.5 months in control and *Cln3*$^{-/-}$ mice. (**b**) Histogram indicating measurements of immunoreactivity area for the corresponding brain regions at 6.5 months in WT controls and *Cln3*$^{-/-}$ mice. No significant changes were detected in any of the brain regions measured. Scale bar= 200 um. Mean ± SEM; ns P>0.05 (T Student test, 5 mice per each genotype and time-point were used).

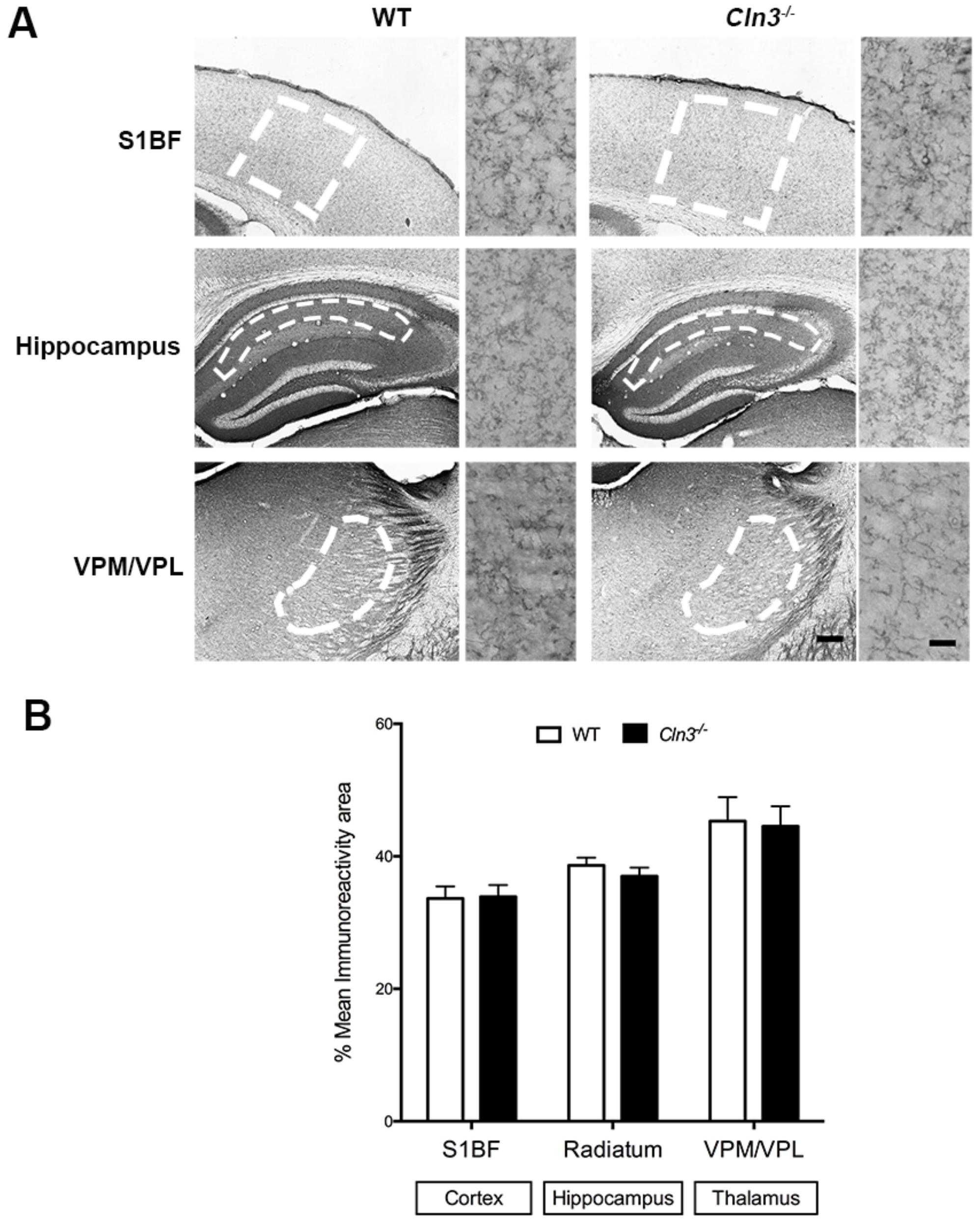

***Fig S2*. The pre-synaptic marker VAMP2 does not show a significant downregulation in thalamic regions at 13 months in *Cln3*$^{-/-}$ mice relative to controls:** (**a**) Representative photomicrographs of coronal sections and high power insets of a thalamic region - the ventral posterior medial/ventral posterior lateral thalamic nucleus (VPM/VPL on the right); its respective cortical projections in the primary somatosensory cortex (S1BF on the left); and the hippocampal stratum radiatum (middle panels) immunostained with VAMP2 **(b)** A histogram showing the corresponding quantification based on VAMP2 immunoreactivity a at 13 months in control and Cln3-/- mice. No significant changes were detected in any of the brain regions measured although the data is suggestive of a decreased signal is evident in both hippocampal and thalamic nuclei. Scale bar= 200 um and 20um for high power insets. Mean ± SEM; (5 mice per each genotype and time-point were used).

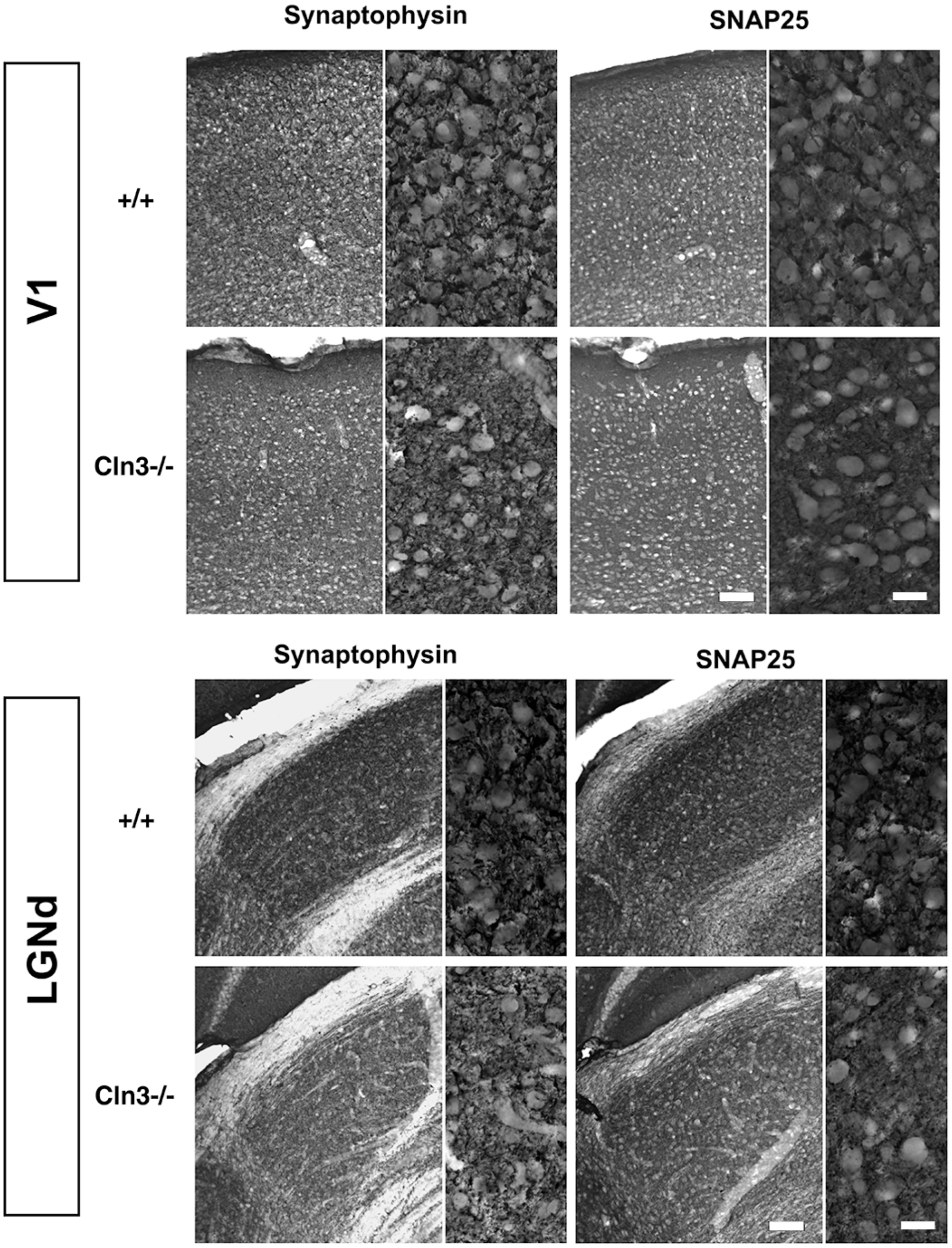

***Fig S3.* The pre-synaptic markers synaptophysin and SNAP25 at 10 months in *Cln3*$^{-/-}$ and controls:** Representative photomicrographs of coronal sections of the primary visual cortex (V1) and the dorsal lateral geniculate nucleus (LGNd) immunostained with synaptophysin and SNAP25 at 10 months in control (+/+) and *Cln3*$^{-/-}$ mice. Scale bar= 300 um and 30um for high power insets.

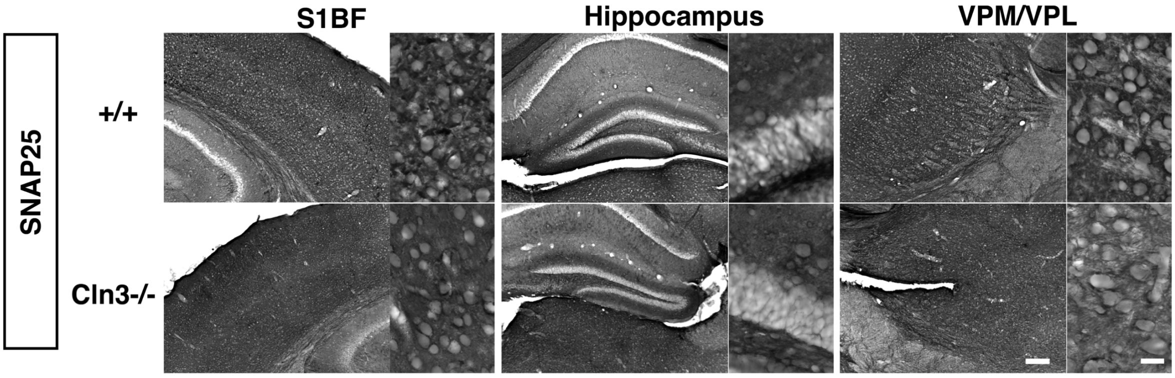

Fig S4. **The pre-synaptic marker SNAP25 at 10 months in *Cln3*$^{-/-}$ mice and controls:** Representative photomicrographs of coronal sections of the primary somatosensory cortex (S1BF), the hippocampus and the ventral posterior medial/ventral posterior lateral thalamic nucleus (VPM/VPL) immunostained with SNAP25 at 10 months in control (+/+) and *Cln3*$^{-/-}$ mice. Scale bar= 200 um and 20um for high power insets.

Valine Degradation

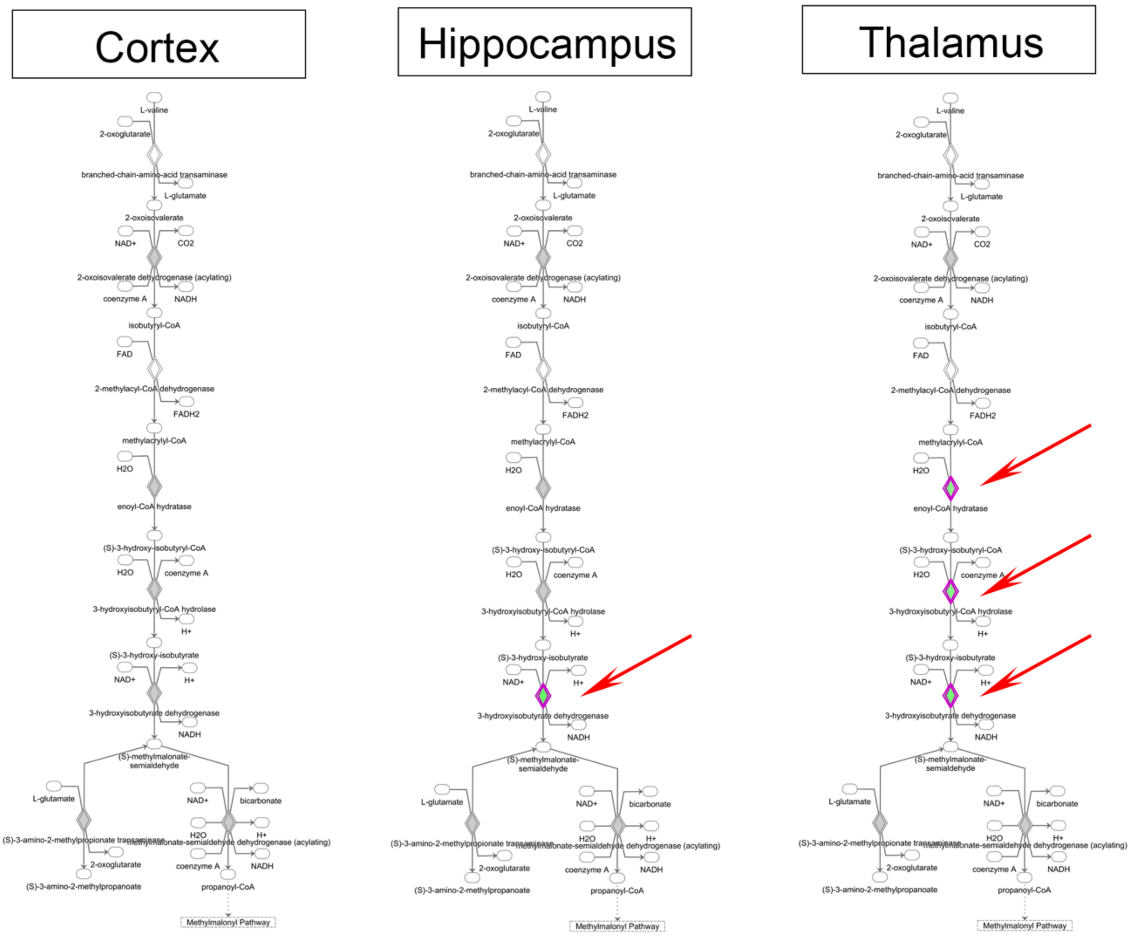

Fig S5. Representation of the valine degradation pathway in cortex, hippocampus and thalamus using IPA. The amount of differentially expressed proteins between *Cln3*$^{-/-}$ and controls correlates with the disease vulnerability status of synapses across brain regions. Red arrows highlight proteins whose expression is disrupted by more than 20%. green = downregulated >20%, grey= change by <20%.

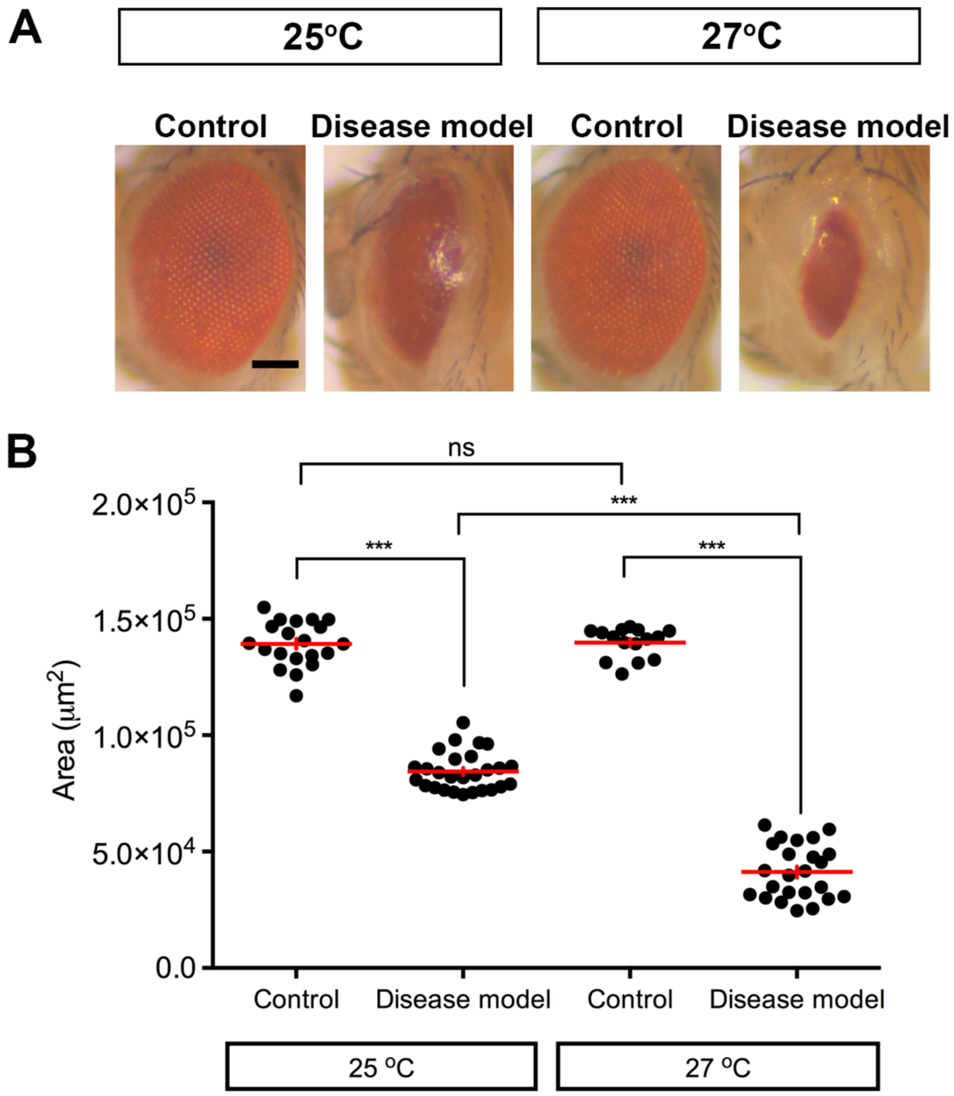

Fig S6. CLN3-induced neurodegeneration in the eye is dose-dependent. (**a**) Representative light microscope images of fruit fly eyes of control line (GMR-GAL4/+) and disease model line (GMR-GAL4/UAS-CLN3) at 25°C and 27°C. The higher the temperature the greater the expression of CLN3. (**b**) Normalized quantification of the eye surface area of these genotypes showing that the higher the dose of CLN3 gene, the greater the effect seen in the eye. Scale bar=100um. (One-way ANOVA with Turkey's multiple comparison post-hoc).

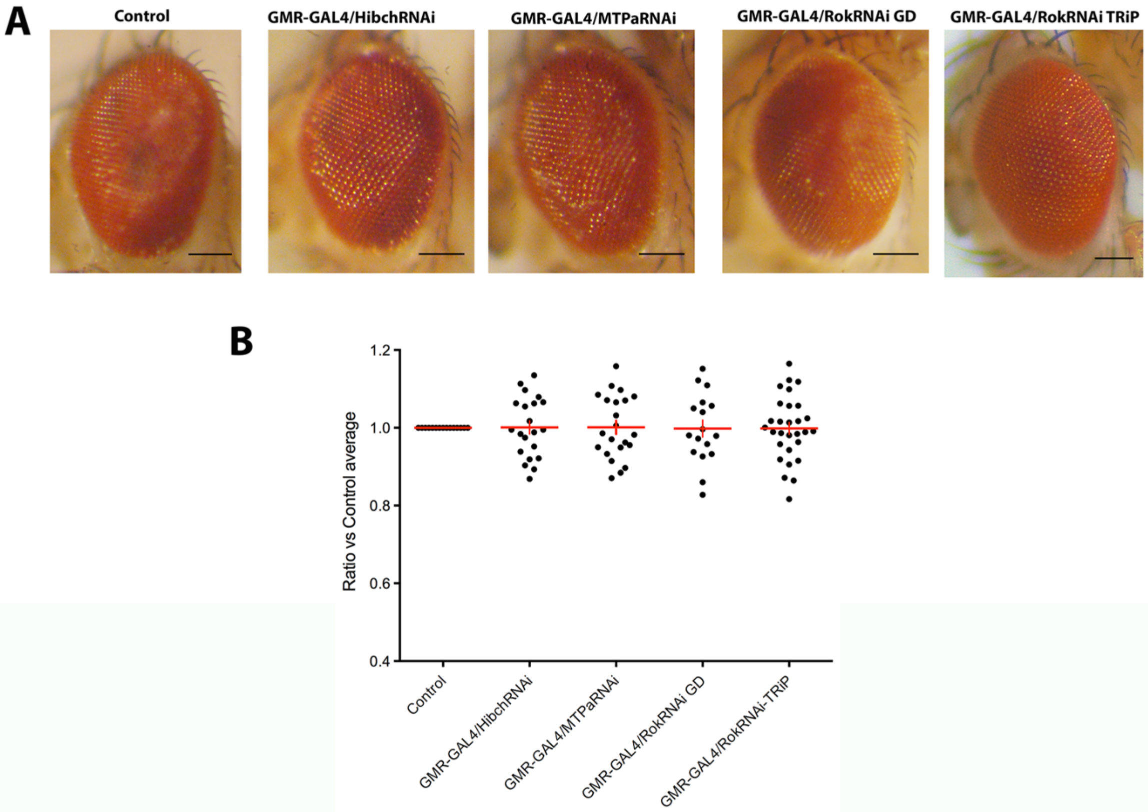

Fig S7. Downregulation of candidates does not give any phenotype in the eye of a WT fly. (**a**) Representative light microscope images of fruit fly eyes of control line, GMR-GAL4/UAS-CLN3, GMR-GAL4/HibchRNAi, GMR-GAL4/RNAiMTPα, GMR-GAL4/RokRNAi GD and GMR-GAL4/RokRNAi TRiP. (**b**) Normalized quantification of the eye surface area of these genotypes showing no statistically significant change when compared to the control line. Scale bar=100um. (One-way ANOVA and Turkey's multiple comparison post-hoc).

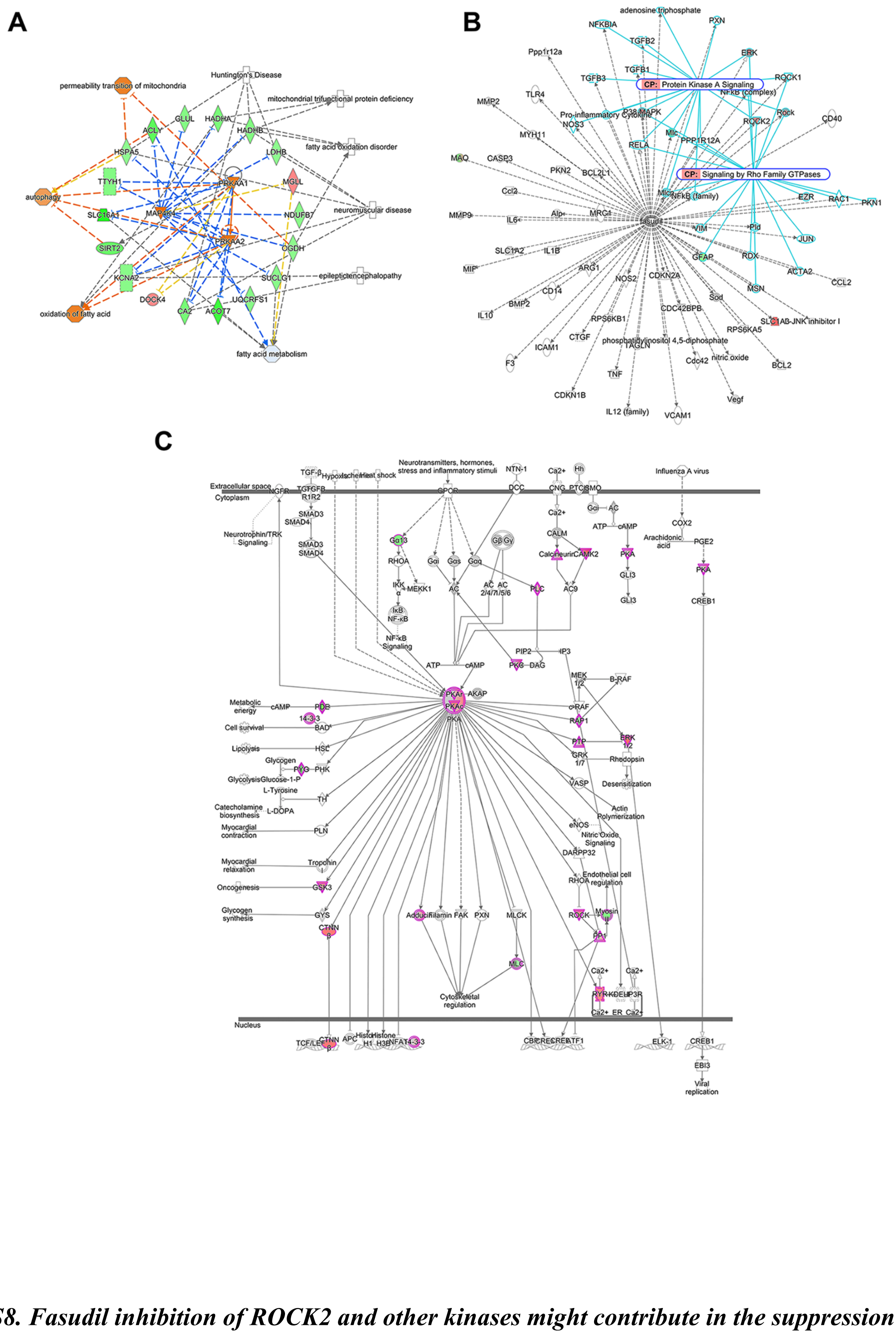

Fig S8. Fasudil inhibition of ROCK2 and other kinases might contribute in the suppression of CLN3 induced degeneration. (**a**) Network representation showing upstream regulator kinases predicted to be activated using IPA upstream regulator analysis that are targeted by

Fasudil. Activation of MAP4K4, PPKAA1 and PPKAA2 (center of the network) impacts on the disrupted expression of proteins such as HADHA, OGDH or SIRT2 (proteins in the thalamic dataset): proteins involved in mitochondria permeability, neuromuscular disease, or autophagy. (**b**) Fasudil interactome highlights the interaction of fasudil with other proteins whose expression is perturbed in thalamic degenerating synapses. Some of these proteins overlap with disrupted pathways such as "Signaling by Rho family GTPases" and "Protein Kinase A signaling"). (**c**) "Protein kinase A signaling" pathway represented by IPA. Upregulation of PKAc (hub in this network), which is also a target of fasudil, impacts in the upregulation of some proteins such as ROCK2 or β-catenin. Orange = predicted activation, blue = predicted inhibition, red = upregulated >20% green = downregulated >20%.

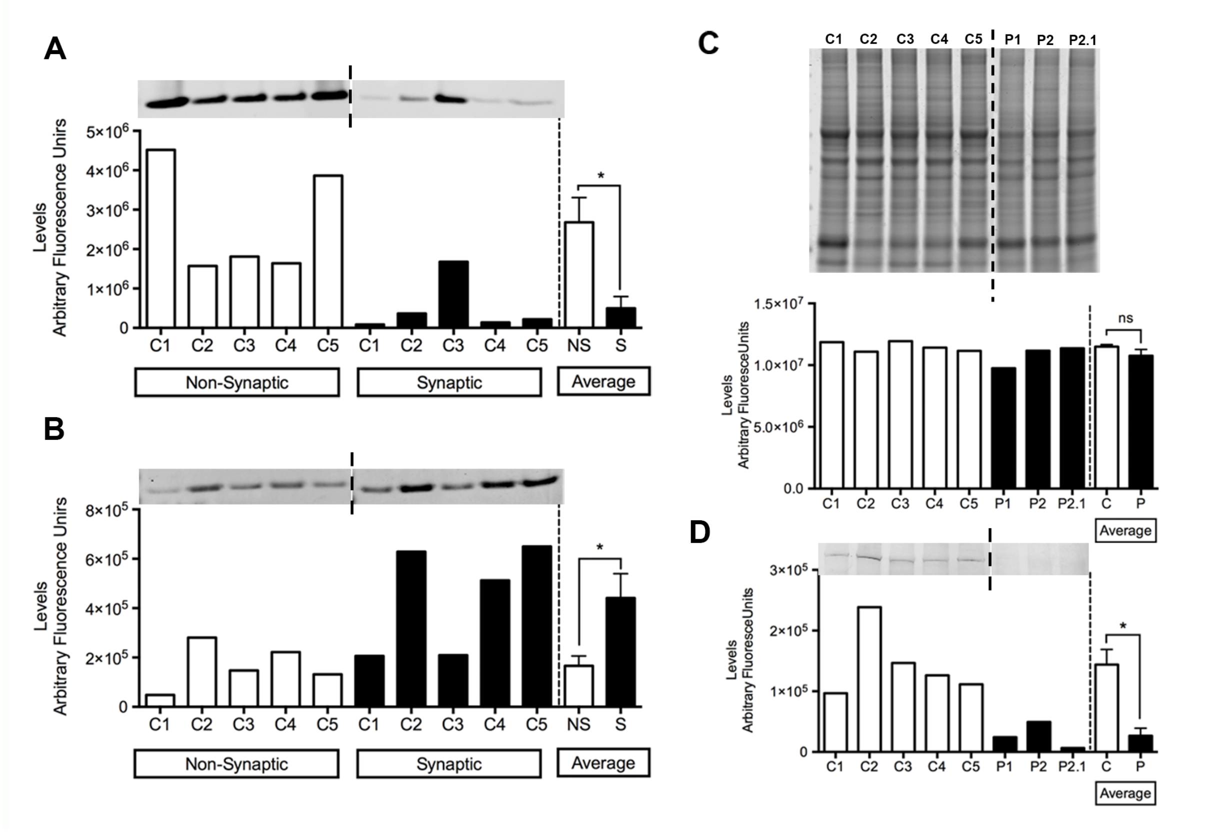

Fig S9. QWB validation of synaptic and non-synaptic isolates from of post mortem brain samples from JNCL patients and controls. (**a&b**) QWB bands and corresponding quantification of protein histone H2Ax (nuclear marker) and synaptobrevin (synaptic vesicle marker) respectively. (**c**) Instant blue staining of total protein as a loading control and validation of integrity of the protein content within synaptic isolates. The banding pattern and quantification showed no obvious protein degradation of the samples. (**d**) QWB bands and quantification of ROCK2 in synaptic fractions showing downregulation of this protein at end stages of the disease. Dotted lines indicate cropping of bands within the same gel.

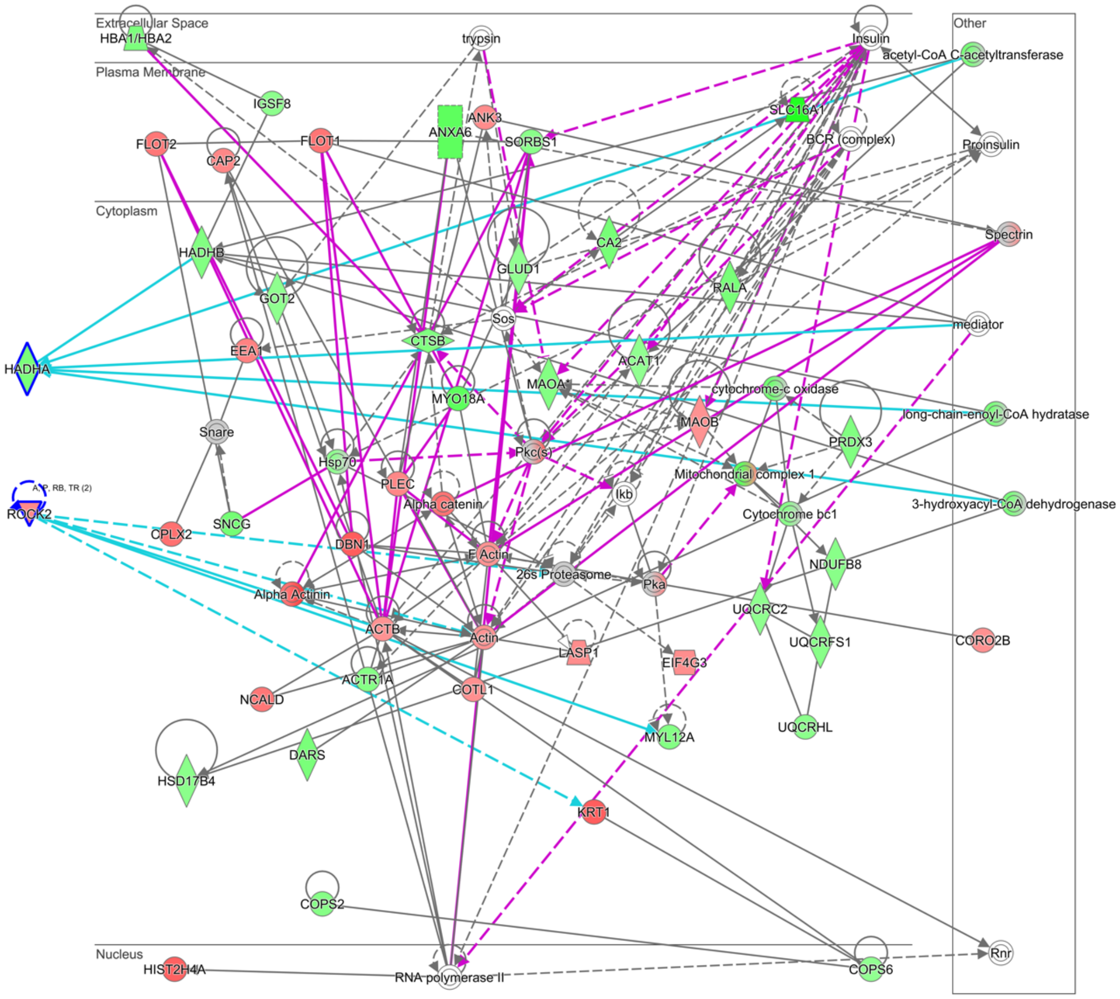

Fig S10. IPA generated network indicating molecular overlaps between HADHA and ROCK2. The valine degradation enzyme (HADHA) and ROCK2 share multiple interactors indicating potential overlapping cascades leading to neurodegeneration. The two merged networks in which HADHA and ROCK2 have a role are "Cell-To-Cell Signaling and Interaction, Drug Metabolism, Energy Production" and "Amino Acid Metabolism, Small Molecule Biochemistry, Cancer" respectively. The interactions between the two networks are highlighted by purple connective lines. HADHA and ROCK2 interactions are highlighted by light blue lines. red = upregulated >20% green = downregulated >20%, grey=change <20%, solid line=direct interaction, dashed line= indirect interaction.

Proteomic mapping of differentially vulnerable pre-synaptic populations identifies regulators of neuronal stability *in vivo*.

Maica Llavero Hurtado[1], Heidi R. Fuller[2+], Andrew M.S. Wong[3+], Samantha L. Eaton[1], Thomas H. Gillingwater[4, 5], Giuseppa Pennetta[4, 5], Jonathan D. Cooper [3,6†] & Thomas M. Wishart [1,5†]*

Supplemental Experimental Procedures

Immunohistochemistry (IHC)

After cryoprotection in a solution containing 30% sucrose, 0.05% sodium azide and 50 mmol/L Tris-buffered saline (TBS), frozen coronal sections of 40 μm were cut with Microm HM430 Sliding Microtome (Microm international, Walldorf, Denmark) as previously described in [102]. Endogenous peroxidase activity was quenched by immersion of floating sections in 1% H_2O_2 in TBS, followed by blocking non-specific protein binding sites in 15% normal goat serum (NGS) (Vector laboratories Cat. *S-1000*). The primary antibody against Synaptophysin (1:100; Upstate), SNAP25 (1:1000, BD Transduction) and VAMP2 (1:500; Chemicon) were incubated overnight at 4°C followed by incubation with the biotinylated secondary antibody incubated for 2 hours (goat anti-rabbit IgG 1:1000, Vector Laboratories Cat. *BA100*). After that, sections were incubated for 2 hours in avidin biotinylated peroxidase-complex incubation (Vectastain® Elite® ABC kit, Vector laboratories Cat *PK-6100*). Visualization was carried out with 3,3'-diaminobenzidine tetra-hydrochloride (DAB) reaction catalysed by H_2O_2 (Sigma). Finally, stained sections were mounted onto Super-frost plus slides and air-dried overnight at room temperature, cleared in xylene and coverslipped with DPX.

Biochemical separation of neuronal compartments

Samples were homogenised in an ice-cold isotonic sucrose solution (0.32 M sucrose, 1 mM EDTA, 5 mM Tris-HCl, pH 7.4). The homogenate was centrifuged in a fixed-angle rotor at 900g for 10 min and the supernatant (S1) was collected. The pellet (P1) was resuspended in sucrose solution and centrifuged again at 900g for 10 min in an effort to free and "trapped" synaptosomes and increase yield. The resulting pellet

(P1') contains 'non-synaptic' material (synapse depleted) i.e. white matter tracts, nuclei, microglia etc. was immediately frozen and stored for later use. The resulting supernatant (S1') was combined with S1 and centrifuged in a fixed angle rotor at 20,000g for 15min. The supernatant (S2) was discarded and the pellet (P2) containing "crude" synaptosomes was washed in a Krebs-like buffer (118.5 mM NaCl, 4.7 mM KCl, 1.18 mM MgCl2, 0.1 mM K2HPO4, 20 mM Hepes, 1.3 mM CaCl2, 10 mM glucose, pH 7.4) then centrifuged at 14,000g for 10min.

Protein extraction for iTRAQ proteomics

"Crude" synaptosomes were resuspended and homogenised in 4 volumes (w/v) of 6M Urea, 2M thiourea, 2% CHAPS, 0.5% SDS and 5% protease inhibitor cocktail (Roche) using a pellet pestle (50 strokes with the pestle, left on ice for 10mins, followed by another 50 strokes with the pestle). The extracts were sonicated briefly and left on ice for 10 minutes, followed by centrifugation at 13,000 x g for 10 minutes at 4°C to pellet any insoluble material. For mass spectrometry analysis, an aliquot of extracted proteins from each mouse (n=6) and each brain region was pooled and precipitated in 6 volumes of ice cold acetone overnight at -20°C. The remaining extracts were stored (un-pooled) at -80°C for quantitative Western blotting validation (QWB). The acetone precipitates were pelleted by centrifugation at 13,000 x g for 10 minutes at 4°C and the supernatant was carefully remove and discarded. The pellets were resuspended in 6M Urea in 50mM TEAB. The protein concentration in each sample was determined using a Bradford protein assay.

Proteomic processing

Sample preparation for mass spectrometry analysis: Reduction, alkylation and digestion steps were performed using the reagents and according to the recommendations in the iTRAQ labelling kit (AB Sciex). The extracts were diluted with 50mM TEAB so that the urea concentration was less than 1M, before the addition of trypsin and overnight incubation at 37°C. The digests were then dried down in a vacuum centrifuge and iTRAQ labelling was carried out according to the instructions in the iTRAQ labelling kit. Each tag was incubated with 85μg of total protein (as determined by a Bradford protein assay).

iTRAQ-labelled peptides were pooled and made up to a total volume of 2.4mL in SCX buffer A (10mM phosphate, pH3 in 20% acetonitrile (Romil, UK)). The pooled peptides (2.4mL) were then separated by strong cation-exchange chromatography (SCX) using a polysulfoethyl A column, 300A, 5uM (PolyLC)) at a flow rate of 400ul/minute. Following sample injection, the column was washed at with SCX buffer A until the baseline returned. The gradient was run as follows: 0-50% SCX buffer B (10mM phosphate, 1M NaCl, pH3 in 20% acetonitrile) over 25 minutes followed by a ramp up from 50% to 100% SCX buffer B over 5 minutes. The column was then washed in 100% SCX buffer B for 5 minutes before equilibrating for 10 minutes with SCX buffer A. Fractions were collected (400ul) during the elution period and dried down completely in a vacuum centrifuge.

Protein identification and quantification by mass spectrometry: The iTRAQ tryptic peptide fractions were each resuspended in 35μl of RP buffer A (2% acetonitrile, 0.05% TFA in water (Sigma Chromasolv plus). Prior to mass spectrometry analysis, fractions were first separated by liquid chromatography (Dionex Ultimate 3000) on a Pepmap C18 column, 200μm x 15cm (LC Packings) at a flow rate of 3μl/minute. Fractions were injected by full-loop injection (20μl) and the order of loading was

randomized to minimise effects from carry-over. The eluants used were: A. 0.05% TFA in 2% acetonitrile in water and B. 0.05% TFA in 90% acetonitrile in water. The gradient was run as follows: 10 minutes isocratic pre-run at 100% A, followed by a linear gradient from 0-30% B over 100 minutes, followed by another linear gradient from 30%-60% over 35minutes. The column was then washed in 100% B for a further 10 minutes, before a final equilibration step in 100% A for 10 minutes. During the elution gradient, sample was spotted at 10 second intervals using a Probot (LC Packings) with α-cyano-4-hydroxycinnamic acid (CHCA) at 3mg/ml (70% MeCN, 0.1% TFA) at a flow rate of 1.2µl/min.

Both MS and MS/MS analysis was performed on the fractionated peptides using an Applied Biosystems 4800 MALDI TOF/TOF mass spectrometer. The mass spectrometer was operated under control of *4000 Series Explorer v3.5.2* software (Applied Biosystems). A total of 1000 shots per MS spectrum (no stop conditions) and 2500 shots per MS/MS spectrum (no stop conditions) were acquired. The following MS/MS acquisition settings were used: 2KV operating mode with CID on and precursor mass window resolution set to 300.00 (FWHM). Peak lists of MS and MS/MS spectra were generated using 4000 Series Explorer v3.5.2 software and the following parameters were used after selective labelling of monoisotopic mass peaks: MS peak lists: S/N threshold 10, Savitzky Golay smoothing (3 points across peak (FWHM)), no baseline correction, MS/MS peak lists: S/N threshold 14; smoothing algorithm: Savitzky Golay, smoothing (7 points across peak (FWHM)).

An automated database search was run using *GPS Explorer v3.6* (AB Sciex). MASCOT was used as the search engine to search the NCBI non-redundant database using the following search parameters: precursor ion mass tolerance of 150ppm,

MS/MS fragment ion mass tolerance of 0.3Da, iTRAQ fragment ion mass tolerance of 0.2Da, the taxonomy was selected as rodents, oxidation of methionine residues were allowed as variable modifications and N-term (iTRAQ), lysine (iTRAQ) and MMTS modification of cysteine residues were set as fixed modifications. Quantification of the iTRAQ peptides was performed using the GPS Explorer v3.6 software. The identification criterion was at least 2 peptides by MS/MS with the most stringent search settings (peptide rank 1 and total ion score confidence intervals of at least 95%). iTRAQ Ratios were normalized using the following formula: iTRAQ Ratio = Ratio / (median iTRAQ Ratio of all found pairs) that was applied in GPS Explorer software.

Quantitative fluorescent western blotting (QWB)

After transfer, membranes were incubated with Odyssey blocking buffer (Li-Cor) prior to incubation with rabbit polyclonal antibodies directed against HIBCH (1:1000, Abcam 101672), HIBCH (1:1000, Proteintech 14603-1-AP), ROCK2 (1:1000, Abcam 71598), ROCK2 (1:1000, Abcam 125025), calretinin (1:1000, Abcam 14689), α-synuclein (1:500, Santa Cruz 7011-R), histone H2A.X (1:1000, Abcam 20669) and mouse polyclonal antibodies to β-catenin (1:1000, BD 610153), synaptobrevin (1:1000, Abcam 11104), overnight at 4°C. Goat anti-rabbit IgG (H+L) 800CW, goat anti-rabbit (680RD) and goat anti-mouse (H+L) (680RD) was applied for 90 minutes at room temperature (1:5000, LI-COR) prior to washing with PBS. Visualisation and quantification was carried out with the LI-COR Odyssey® scanner and software (LI-COR Biosciences). Blots (and gels) were imaged using an Odyssey Infrared Imaging System. Scan resolution of the instrument ranges from 21 to 339 μm, and in this study

blots (and gels) were imaged at 169 μm. Quantification was performed on single channels with the analysis software provided as previously described [2,91,92,94].

Proteins for proteomic validation were chosen by a combination of their previous implication in neurodegeneration processes, and on their magnitude of change [2,17,95,103]. For example, β-catenin and a α-synuclein are good representatives of validated large changes; HIBCH and ROCK2 are representative of validated moderate changes, and calretinin is a good example of an unchanged protein from the data set (Fig 4B-G).

Biolayout Express

This software was developed in the Roslin Institute (The University of Edinburgh) and further information on the use of this program and free access can be found here: http://www.biolayout.org. Analysis of cortical, hippocampal and thalamic synaptic proteomic data using *BioLayout* allowed clustering of proteins in order to correlate their expression profile to the differentially vulnerable synaptic population across brain regions (Fig 3D&E) [45]. In this display each sphere represents an individual protein and its colour and proximity to its neighbour indicates the similarity in protein expression. Clusters (groupings of proteins delineated by colour) can be further analysed for cell type or functional association with other *in silico* tools such as enrichment analysis.

Ingenuity Pathway Analysis (IPA)

IPA dynamically generates networks of gene, protein, small molecule, drug, and disease associations on the basis of "hand-curated" data held in a proprietary database. More than 90% of the information in this database is "expert-curated" and is drawn from the full text of peer reviewed journals. Less than 10% of interactions have

been identified by techniques such as natural language processing. Canonical pathways function was used for the identification of known pathways within this dataset. IPA contains a library of biological pathways published in the literature that are ranked by significance of the association between the dataset and the canonical pathway. The significance is defined by two parameters: (1) the ratio of the number of proteins from the dataset that map on the pathway divided by the total number of genes within the canonical pathway and, (2) a P value calculated using Fischer's exact test that determines if the probability of association between proteins in the dataset and in the pathway are due to chance. Prediction activation scores (z-score) is a statistical measure of the match between an expected relationship direction and the observed protein expression. Positive z-score indicates activation (orange) and negative z-score indicate inhibition (blue) [98]. 1.2 Fold-change threshold filter was applied in IPA to the dataset analysed and only experimentally observed interactions were selected for this analysis. Further information on the computational methods implemented in IPA can be obtained from Ingenuity Systems. Available at http://www.ingenuity.com/.

Proteomic mapping of differentially vulnerable pre-synaptic populations identifies regulators of neuronal stability *in vivo*.

Maica Llavero Hurtado[1], Heidi R. Fuller[2+], Andrew M.S. Wong[3+], Samantha L. Eaton[1], Thomas H. Gillingwater[4, 5], Giuseppa Pennetta[4, 5], Jonathan D. Cooper [3,6†] & Thomas M. Wishart [1,5†]*

<u>Raw western blot membrane images for cropped bands in main figures</u>

Fig 4. Panel B Beta-catenin

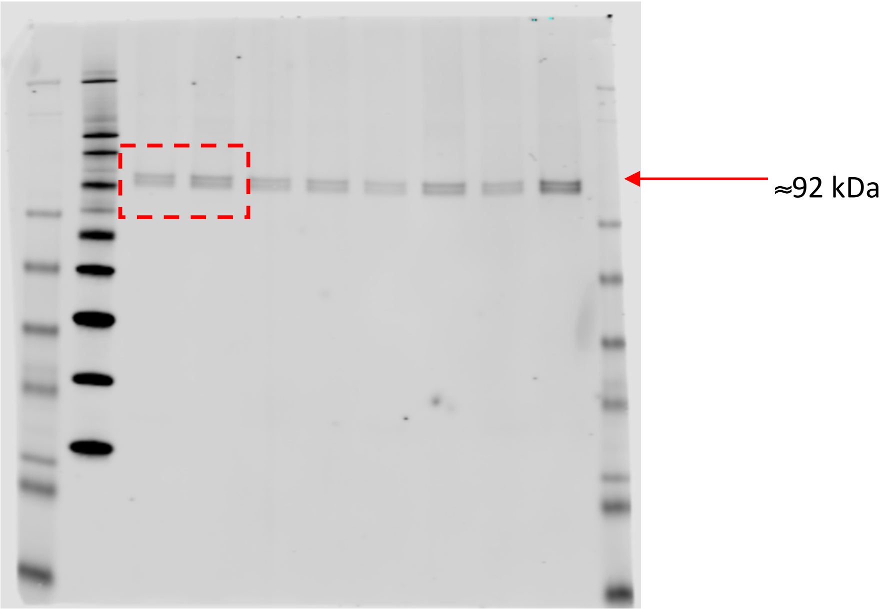

Fig 4. Panel B and Fig 7 Panel B, Rock2

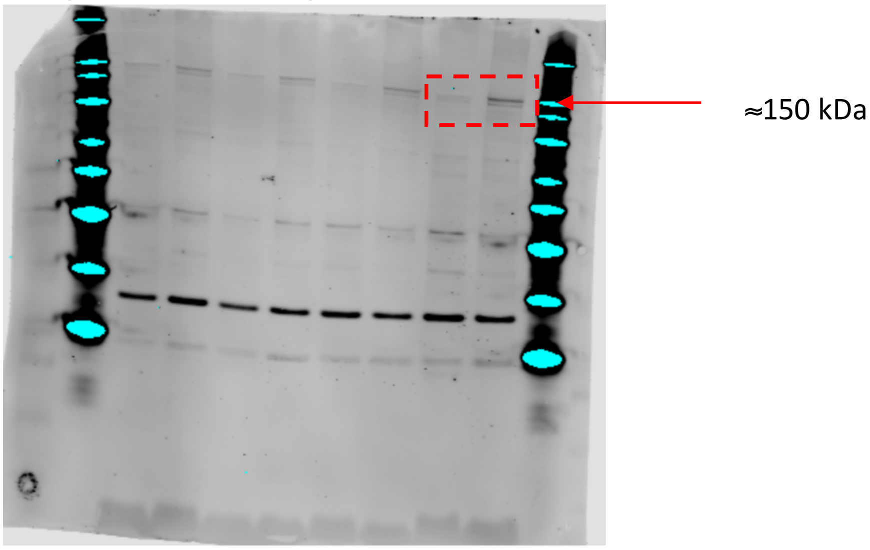

Fig 4. Panel B Calretinin

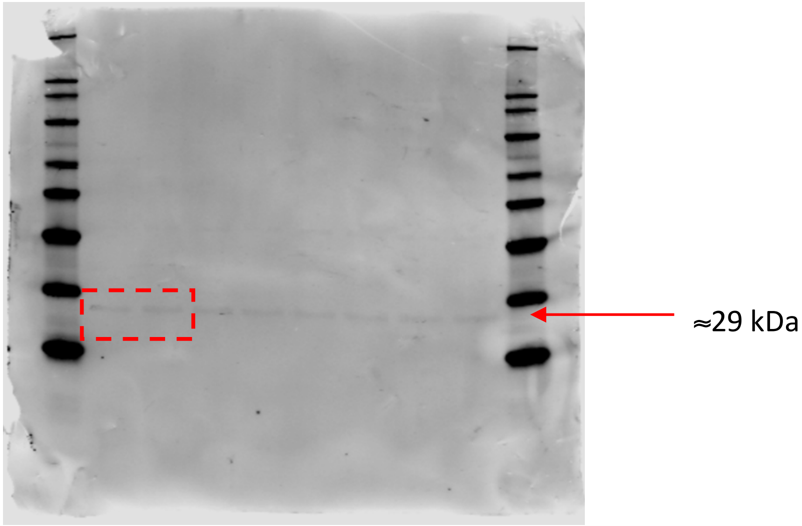

Fig 4. Panel B Alpha-synuclein

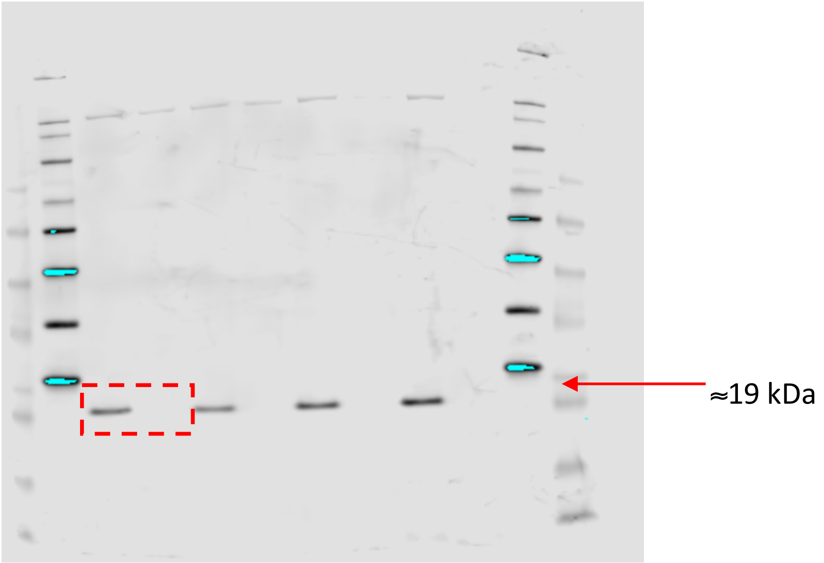

Fig 4. Panel B and Fig 6, Hibch Panel B (synaptic)

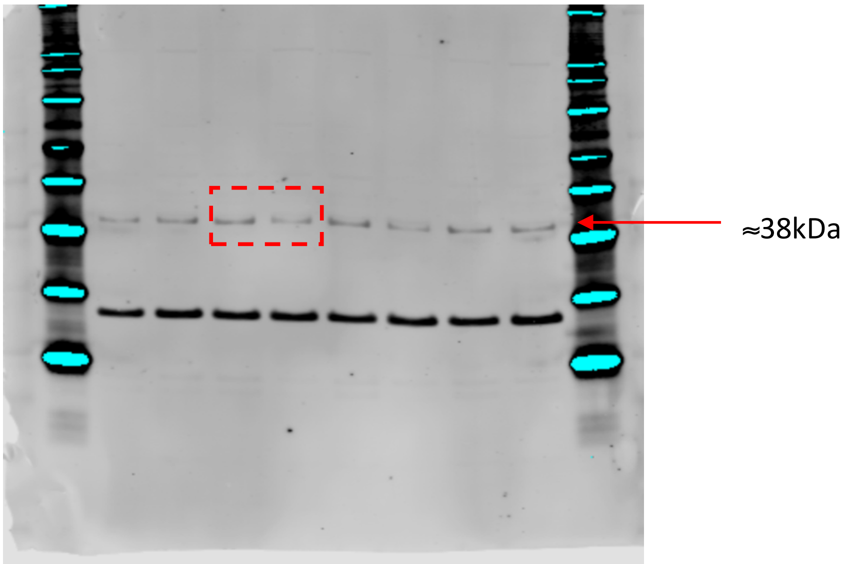

Fig 6. Panel B Hibch Non-synaptic

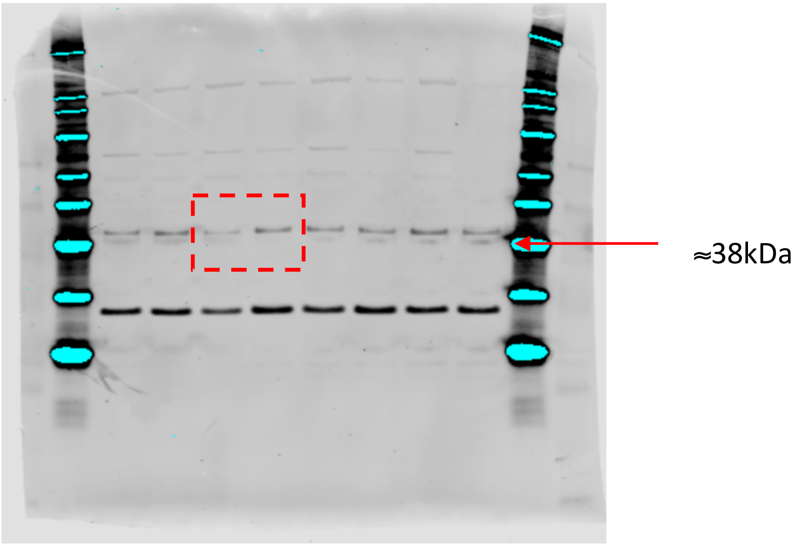

Fig 6. Panel C, Hibch human synaptic

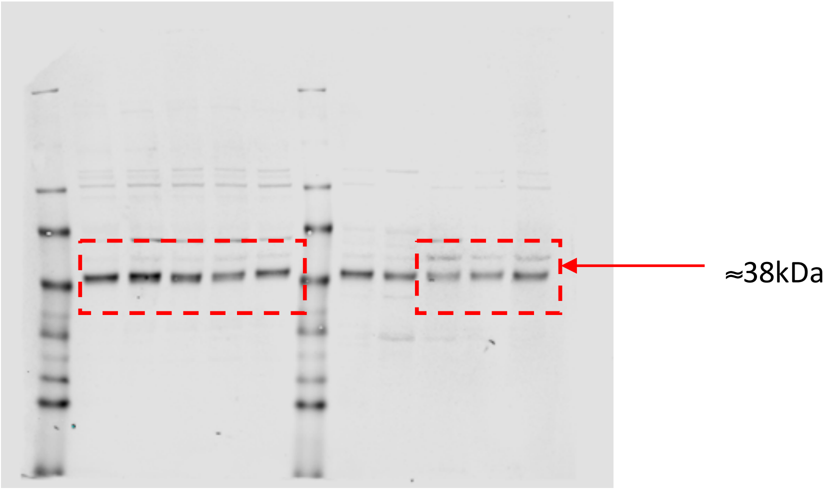

Fig 7. Panel B, Rock2 (Non-synaptic)

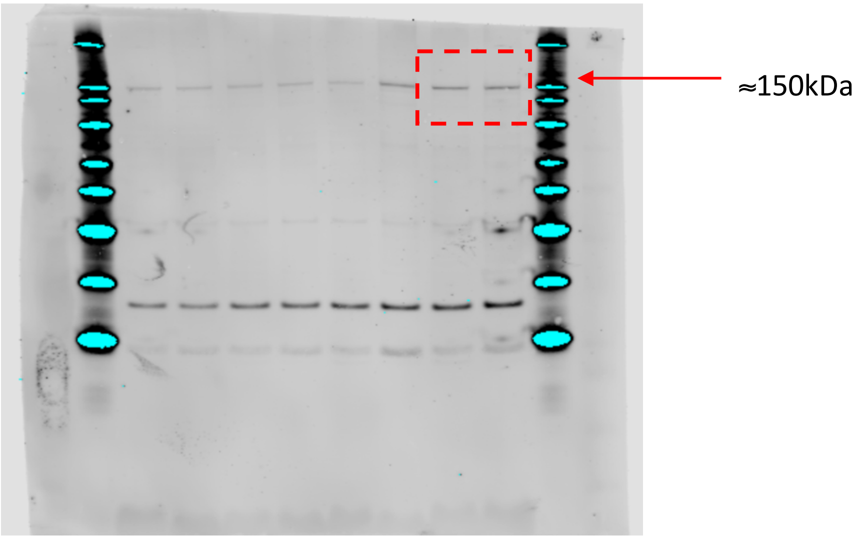

Supplement: Supplementary file 1 — Supplementary Information file [file 41598_2017_12603_MOESM1_ESM.pdf]
